# Stochastic Impact Electrochemistry of Alkanethiolate‐Functionalized Silver Nanoparticles

**DOI:** 10.1002/smll.202410306

**Published:** 2025-03-13

**Authors:** Lennart J. K. Weiß, Marta Nikić, Friedrich C. Simmel, Bernhard Wolfrum

**Affiliations:** ^1^ Physics of Synthetic Biological Systems (E14) Department of Bioscience School of Natural Sciences Technical University of Munich 80333 München Germany; ^2^ Neuroelectronics Munich Institute of Biomedical Engineering School of Computation Information and Technology Technical University of Munich 80333 München Germany

**Keywords:** redox activity, self‐assembled monolayer, silver nanoparticles, stochastic collision electrochemistry, surface functionalization

## Abstract

This study uses single‐impact experiments to explore how the nanoparticles’ surface chemistry influences their redox activity. 20 and 40 nm‐sized silver nanoparticles are functionalized with alkanethiol ligands of various chain lengths (*n* = 3, 6, 8, and 11) and moieties (carboxyl ─COOH / hydroxyl ─OH), and the critical role of the particle shell is systematically examined. Short COOH‐terminated ligands enable efficient charge transfer, resulting in higher impact rates and fast, high‐amplitude transients. Even elevated potentials fail to overcome tunneling barriers for ligand lengths of *n* ≥ 6 and risk oxidizing the electrode, forming an insulating layer. Electrostatic interactions play a key role in governing reaction dynamics. In general, particles with a COOH‐group exhibit higher impact rates and current amplitudes in KCl than those with an OH‐group. This effect is more pronounced for 40 nm‐sized particles; although, they rarely oxidize completely. The influence of electrolyte composition—concentration, pH, and a biologically relevant electrolyte—reveals that its impact on the redox activity can be as critical as that of the particle shell, with both determining particle adsorption and electron tunneling. These findings provide insights into the complex interdependencies at the electrode–particle–electrolyte interface, aiding the design of custom redox‐active (silver) nanoparticles for ultrasensitive electrochemical sensing.

## Graphical abstract

This study investigates the influence of alkanethiol monolayer characteristics—chain length, functional end groups, and particle size—on the redox activity of silver nanoparticles via single‐impact experiments. The redox activity decreases with increasing chain length; while the functional end group and the electrolyte composition play a predominant role by significantly modulating particle adsorption.

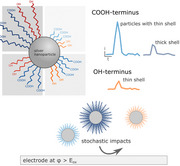

## 1 Introduction

In the past two decades, single‐impact electrochemistry has emerged as a rapidly growing field of research, offering new insights into the complex interactions of single (redox‐active) entities with the electrode–electrolyte interface.^[^ ^1^, ^2^ ^]^ This field studies perturbations—conveniently discrete current signals—arising from colliding nanoparticles with a (biased) electrode of µm‐size. The collisions of metallic nanoparticles are typically associated with spike‐shaped or step‐like current perturbations (corresponding to either electro‐dissolution, for example, in the case of silver (Ag), nickel, copper, or catalytic activation for gold, platinum, and palladium particles), which are distinct from the electrochemical background signal.^[^ ^3^, ^4^, ^5^, ^6^ ^]^ This “digital” feature has been employed recently to detect very dilute species by using redox‐active nanoparticles as labels.^[^ ^4^, ^7^, ^8^, ^9^ ^]^ Impact‐based sensing strategies, however, require a profound understanding of the electrode–nanoparticle interactions, with the particle corona playing a crucial role.^[^ ^3^, ^10^, ^11^, ^12^, ^13^, ^14^ ^]^ Particle collision experiments were shown to be very sensitive to the functionalization of or impurities at the electrode surface,^[^ ^14^, ^15^ ^]^ rendering this technique particularly effective for examining the role of nanoparticle surface coatings.^[^ ^16^, ^17^ ^]^ We here explore this aspect in detail and aim to provide general insights for a rational design of redox‐active labels, which can be, in particular, an intricate task for silver nanoparticles.^[^ ^10^, ^18^, ^19^, ^20^, ^21^ ^]^ The nanoparticle corona must support colloid stability, efficient detection, and additional specificity in electrochemical sensing.^[^ ^22^, ^23^, ^24^ ^]^ In general, the detectability of a nanoparticle depends on its dynamic interactions with the electrode. Therefore, modifications to the electrode or the nanoparticle surface, as well as, changes in the bias potential or the buffer conditions are expected to significantly influence the particle–electrode interactions.^[^ ^11^, ^14^, ^25^, ^26^ ^]^ In this work, we systematically investigate how various characteristics of the particle coating, including chain length, terminal group, and nanoparticle size, influence the particle redox activity. To provide general insights, we solely focus on alkanethiol molecules with convenient terminal groups, which can be employed as co‐ligands in mixed assemblies. We modify silver nanoparticles with *n*‐alkanethiolates of various chain lengths, *n* = 3, 6, 8, 11, and end groups (carboxyl ─COOH / hydroxyl ─OH) and perform impact recordings, as well as, optical measurements in different background electrolytes. We also assess the ambiguous role of the applied oxidation potential, which not only alters the likelihood of oxidation but also affects the electrode surface and particle transport. Further, we analyze how the nanoparticle size impacts on the detection yield by comparing results for 20 and 40 nm‐sized particles. Last, we explore changes in particle behavior under varying buffer compositions.

Our key findings reveal that the terminal group of the particle shell governs the interfacial dynamics, with electrostatic interactions playing a critical role in facilitating efficient charge transfer. This effect scales with particle size as larger particles (e.g., 40 nm) exhibit stronger electrostatic effects due to their greater contact area. In addition, the thickness of the particle shell is essential and ligand lengths *n* > 6 drastically impede charge transfer. While increasing the electrode potential enhances the number of collisions for particles with very thin shells (*n* = 3), thicker shells remain impervious even at higher potentials. We also illustrate that the electrolyte solution plays a pivotal role not only in governing particle adsorption and residence time within the tunneling region but also in shaping the energy landscape and controlling the dissolution of the reaction products. Here, higher chloride concentrations enhance the redox activity of COOH‐terminated particles but show no effect on OH‐terminated particles. Interestingly, variations in pH or the presence of diverse anionic species significantly influence particle–electrode interactions, which results in contrasting behavior of COOH‐ and OH‐terminated particles.

This work extends the findings from Lu et al.,^[^ ^14^ ^]^ Dery et al.,^[^ ^16^ ^]^ and Krause et al.,^[^ ^15^ ^]^ who investigated silver nanoparticle collisions at alkanethiol‐covered electrodes. We further complement the research on electrocatalytic impacts, where nanoparticle‐enabled electron transfer across an insulating layer had been studied.^[^ ^27^, ^28^, ^29^, ^30^ ^]^ Overall, this work expands the existing knowledge on the physico‐ and electrochemical properties of nanoparticles using athiolate self‐assembled monolayers.^[^ ^10^, ^12^, ^26^, ^31^, ^32^, ^33^, ^34^, ^35^, ^36^, ^37^, ^38^, ^39^, ^40^, ^41^ ^]^

## 2 Results and Discussion

While previous fundamental studies on particle–electrode interactions have often relied on chemically modified electrodes and as‐is particles,^[^ ^14^, ^15^, ^42^ ^]^ applied electrochemists independently utilized customized nanoparticle coatings to develop ultrasensitive impact‐based sensors.^[^ ^7^, ^9^ ^]^ To bridge this gap, we systematically examined how the particles’ coatings modulated their redox activity. Specifically, we modified 20 and 40 nm‐sized nanoparticles with alkanethiol ligands of different lengths *n* = 3, 6, 8, 11, and terminal group (─COOH / ─OH), leading to 16 different particle variants. The redox activity was analyzed in terms of impact frequencies and the evaluation of the current transients. With this approach, we aim to reveal and better understand the (critical) role of the nanoparticles’ surface chemistry.

### 2.1 Existing Model Frameworks for Particle Redox Activity

**Figure 1:**
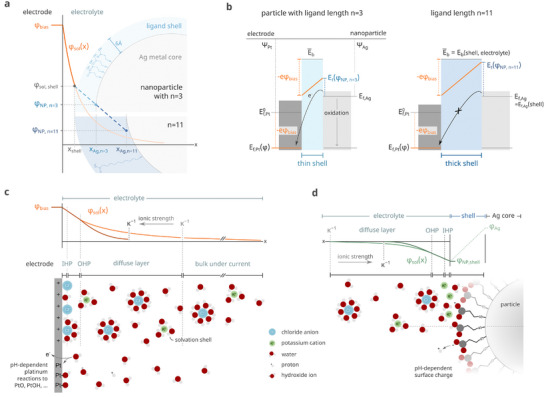
Oxidative nanoparticle impacts require activated electron tunneling through the nanoparticle shell and the adjacent electrochemical double layer. Variations in electron tunneling arise from separating distances and effective tunneling barriers *E*_t_(φ_NP_). a) The thickness of the ligand shell affects the potential present at the Ag surface. Particles with thinner shells experience a higher effective potential φ_NP_ at their metal core due to the reduced distance to the electrode. b) Particle oxidation as a process of Fermi level equilibration across a tunneling barrier of average height ${\bar{E}}_{b}$ for thin and thick ligand shells. The reaction occurs, if the energy of the electron inside the platinum electrode *E*_f,Pt_(φ) is lower than its energy inside the nanoparticle *E*_f,Ag_, and the effective energy barrier height *E*_t_(φ) for tunneling across the ligand shell is in the range of *k*_B_ *T*. The spatial structure of the ligand (e.g., odd or even number of C‐atoms, tilt angle with Ag facets, terminal groups) and its assembly can further influence ${\bar{E}}_{b}$ and the electronic state of the Ag atoms *E*_f,Ag_. The different metals are characterized by their work functions, ψ_Pt_ and ψ_Ag_. c) Electrochemical double layer and potential distribution φ_sol_(*x*) (orange) at the working electrode under a positive bias potential and in KCl solution. Inner (IHP) and outer Helmholtz planes (OHP) define well‐ordered layers of screening anions and solvated cations. The electrolyte's ionic strength determines the extent of the diffusive layer κ^−1^. Higher ionic strengths compress the double layer, resulting in steeper potential gradients near the electrode (red). The electrode potential can also drive additional reactions (e.g., platinum oxidation and oxygen evolution reaction), leading to a persistent faradaic background current. d) Electrochemical double layer and potential distribution φ_sol_(*x*) (around a silver nanoparticle with COOH‐terminated ligands for lower (green) and higher ionic strengths (dark green). The surface charge of the terminal groups is pH‐dependent, thereby affecting the potential distribution around the particle.

To better understand the critical interdependencies in our experimental data, we begin by identifying and summarizing the key factors that determine particle redox activity. First and foremost, the collision frequency is limited by the diffusive (and electrophoretic) particle flux from the bulk toward the electrode. Once a particle reaches the electrode, its electrochemical conversion involves multiple steps, including adsorption, tunneling, transfer, and the dissolution of the remaining reaction products, with the electrode's potential primarily dictating the reaction dynamics.^[^ ^43^, ^44^ ^]^ In solution, the electrode potential decays exponentially in the diffuse layer according to Poisson–Boltzmann (see **Figure** **1**a,c) but might be altered in the presence of the nanoparticle.^[^ ^14^ ^]^ For an approaching particle to oxidize, its electrons must tunnel through dielectric material, as illustrated in Figure 1b.^[^ ^14^, ^43^ ^]^ The driving force for the electron transfer (Fermi level equilibration) is determined by the energy difference between electron states in the nanoparticle and the electrode. The kinetics (on‐rate of the forward pathway) is governed by the height of the energy barrier separating the two electron states. After losing their electrons, the remaining Ag^+^ ions of the nanoparticle typically react further with anionic electrolyte species, forming soluble products.^[^ ^44^, ^45^, ^46^, ^47^ ^]^ The overall rate constant in 1/s can be considered as a chain of reaction substeps:

(1) $$\frac{1}{k_{\mathrm{ox}}}=\frac{1}{k_{\mathrm{ad}}}+\frac{1}{k_{\mathrm{et}}}+\frac{1}{k_{t}}+\frac{1}{k_{\mathrm{ds}}}$$

where *k*_ad_ represents the rate of particle adsorption, *k*_et_ the heterogeneous electron transfer, *k*_t_ the electron tunneling through the ligand shell (and solution), and *k*_ds_ reflects the dissolution process.

Typically, *k*_et_ is modeled as a classical Butler–Volmer relation with an exponential dependence on the overpotential φ_bias_ − *E*_eq_. The electronic coupling between the particle and the electrode can be described by the Simmons model. This model treats the interaction as exponentially decaying tunneling between two dissimilar electrodes separated by a homogeneous insulating film, assuming that there is no leakage conduction through pinholes.^[^ ^48^, ^49^ ^]^ The adhesion of the particle to the electrode *k*_ad_ is most often neglected but can be reasoned by density functional theory calculations.^[^ ^11^ ^]^ A model for the dissolution process connects the duration of the current peak to the flux of anions.^[^ ^44^, ^46^, ^50^ ^]^ Here, it is shown that for high overpotentials at the electrode, the Ag oxidation becomes diffusion‐limited as the flux of co‐reacting anions (e.g., Cl^−^ or Br^−^) is bounded by their diffusion constant, whereas for lower overpotentials, the electron tunneling and transfer are the critical steps.^[^ ^28^, ^44^, ^47^ ^]^

The electrolyte, however, not only affects dissolution but also plays a critical role in activated electron tunneling. While the Simmons model in Figure 1b assumes a homogeneous dielectric layer with averaged properties, in reality, the electron must traverse two distinct layers, namely the alkanethiolate shell and the electrolyte. The energy barrier imposed by the alkanethiolate molecules is relatively constant, whereas the energy landscape created by the electrolyte is dynamic and strongly depends on the particle's position, relative to the electrode and the characteristics of the supporting electrolyte. Figure 1c,d illustrate that both the electrode and the particle feature an electrochemical double layer, attracting ions from the solution to screen their surface charges. At the interface, static adlayers of screening and solvated ions form the inner and outer Helmholtz planes. Beyond these planes lies a diffuse layer, whose spatial extent, given by the Debye length κ^−1^,  is determined by the electrolyte's composition and concentration. High ion concentrations compress the diffusive layer and create a steep potential gradient near the electrode (and the nanoparticle; see Figure 1c,d).

To better account for the complex role of the electrolyte in particle oxidation, it is necessary to revisit the assumptions underlying Equation (1). While this framework provides valuable qualitative insights, it oversimplifies the interplay between tunneling and activated charge transfer by treating them as sequential processes. In reality, these processes occur simultaneously and require a rigorous integration of thermodynamic principles with quantum mechanics.^[^ ^51^, ^52^, ^53^, ^54^, ^55^ ^]^ The Marcus–Hush formalism^[^ ^56^, ^57^ ^]^ provides a more comprehensive framework for electron transfer, linking the reaction rate k′_et_ to the Gibbs free energy change Δ*G*, the electronic coupling |*V*|^2^, and the reorganization energy λ via:
(2)

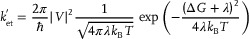

where

(3) $$\Delta G=\Delta G^{0}-nF\left(\varphi_{\mathrm{bias}}-\varphi_{\mathrm{sol}}\left(x\right)\right)$$

with Δ*G* ^0^ as the standard Gibbs free energy change for the reaction, *n* the number of electrons transferred, and F the Faraday's constant. The reorganization energy λ represents the energy required to adjust the structures of the reactants, surrounding ions, and solvent molecules for electron transfer. As shown in Equation (2), several aspects must align for efficient electron transfer, with the electrolyte playing a decisive role in determining Δ*G* and λ. For example, a high Δ*G* requires a steep potential gradient φ_bias_ −  φ_sol_(*x*), facilitated by high ionic strength (see Figure 1a,c). The reorganization energy λ includes inner‐sphere contributions (λ_inner_) from molecular distortions within the reactants and outer‐sphere contributions (λ_outer_) from solvent and electrolyte rearrangements. A compressed double layer aligns ions closer to the electrode, lowering λ_outer_ by minimizing solvent rearrangements; while, the strong electric field reduces at the same time λ_inner_  by stabilizing the reactants and minimizing bond distortions. Thus, high electrolyte concentrations may initially appear universally favorable as they compress the double layer κ^−1^, enhance Δ*G*, and reduce λ. However, the electronic coupling |*V*|^2^ relies on a significant overlap between the wavefunctions of the electron in the particle and the electrode, which is favored by a small potential difference φ_bias_ −  φ_sol_(*x*).^[^ ^53^, ^55^ ^]^ In contrast, a steep potential gradient at the interface misaligns these states, reducing coupling efficiency and shifting the optimal electron transfer distance closer to the electrode.

Given these interactions, the supporting electrolyte shapes an energy landscape with narrow regions where both the driving force and tunneling conditions are fulfilled.^[^ ^58^ ^]^ Thus, as a particle approaches the electrode surface, it traverses this energy landscape, where the conditions for conversion change drastically. Yet, “approaching” does not necessarily imply unidirectional motion but rather a biased diffusion process. Therefore, it might be possible that particles enter the tunneling zone but are then able to escape again before they become (fully) oxidized.

To experimentally test these theoretical insights, we systematically varied key parameters such as ligand length, terminal group, and particle size. The following sections analyze how these factors shape particle–electrode interactions. Finally, we highlight the electrolyte's critical role in modulating reaction dynamics.

### 2.2 Particle Redox Activity and Ligand Length

**Figure 2:**
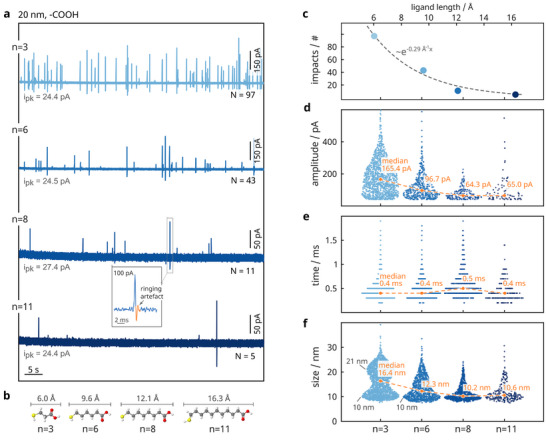
Effect of ligand length on the particle redox activity. a) Exemplary raw current recordings from a single electrode for 30 pM of 20 nm‐sized nanoparticles with carboxyl‐terminated shells of different chain lengths *n* measured in 30 mM KCl. The applied potential was set to 800 mV versus Ag/AgCl, resulting in a peak‐to‐peak current noise *i*_pk_ ≈ 25 pA during all measurements. *N* indicates the number of identified impacts observed during the experimental time shown in the trace. b) The particle shell thickness was approximated by the linear extension of the ligands (*n* = 3, 6, 8, and 11 C‐atoms), neglecting the tilt angle of the molecular axis relative to the Ag(111) lattice.^[^ ^59^, ^60^, ^61^ ^]^ c) Effect of ligand length on the number of nanoparticle collisions. The number of collisions, extracted from the raw data in (a), decayed exponentially with β = −0.29 Å^−1^ (*R* ^2^ = 0.983) as a function of ligand length, assuming ideal monolayers and neglecting any additional gap between the particle and the electrode. d–f) The subpanels show swarm plots of characteristic values derived from all identified current peaks as a function of ligand length *n*, including maximum current amplitudes (d), time durations of particle collisions (e), and estimated particle sizes (f). The statistical analysis is based on data obtained from 120‐second recordings across 15 electrodes for each ligand length. The distribution of particle sizes in (f) was deduced from the injected charge upon each collision, assuming a spherical particle geometry. Note that subpanels (c–f) share the same categorial *x*‐axis.

We first studied the influence of the ligand length and performed experiments under typical conditions: 30 pM of carboxyl‐terminated 20 nm‐sized particles were oxidized in 30 mM KCl solution at a constant potential of 800 mV versus Ag/AgCl. Throughout the manuscript, all potential values are reported with respect to the Ag/AgCl reference electrode. Exemplary current traces for particles with chain lengths of *n* = 3, 6, 8, and 11 are depicted in **Figure** **2**a. Obviously, the thickness of the particle shell has a crucial influence on the impact recordings as the number of impacts decreases with increasing ligand length. For instance, we recorded with a single electrode, 97 current peaks for particles with a shell of chain length *n* = 3, 43 spikes for *n* = 6 particles and just 5 spikes for *n* = 11. This observation aligns with the findings of Lu et al.,^[^ ^14^ ^]^ who also reported a decrease in redox activity with increasing monolayer thickness on the electrode.

In both cases, this behavior can be attributed to a geometrical effect. The electrode potential drops in solution exponentially with distance from the electrode (given by the linearized Poisson–Boltzmann Equation); see Figure 1a,c. Thus, we assume that particles with thinner shells experience at their minimum distance a higher effective (over)potential φ_NP_ at the metal–monolayer interface compared to particles with thicker shells. To determine the monolayer thickness of the different particles, we used the linear extension of the molecules as a reference, as illustrated in Figure 2b. With the potential in solution following φ(*x*) ∝ e^−*x*^; while, the rate of charge transfer is governed by an Arrhenius‐relationship $k_{\mathrm{et}}\propto e^{\varphi \left(x\right)-E_{\mathrm{eq}}}$ (consistent with classical Butler‐Volmer kinetics and the underpinnings of Marcus theory), we understand the drastic changes in Figure 2a because *x*_Ag,*n* = 3_ < *x*_Ag,*n* = 11_; ultimately resulting in *k*_et,thin_ ≫ *k*_et,thick_. Further, the rate of electron tunneling across a dielectric layer can be modeled as:

(4) $$k_{t}\propto e^{-\beta x}$$

where *x* is the separation distance between the Ag atoms and the electrode surface.^[^ ^48^, ^49^, ^62^, ^63^, ^64^ ^]^ The decay factor reflects the mean barrier height of the medium via $\beta \propto \sqrt{{\bar{E}}_{b}}$ and is typically in the range of 1Å^−1^.^[^ ^48^, ^49^, ^64^, ^65^, ^66^, ^67^, ^68^ ^]^ In our case, the approaching particle was separated from the electrode by a combination of electrolyte and the alkanethiol shell; see Figure 1a. The critical dependence of the particles’ redox activity on the electron transport across the dielectric monolayer; and thus, on the height of the tunneling barrier, was illustrated by Bard and co‐workers.^[^ ^17^ ^]^ They showed that nanoparticles could entirely lose their electrocatalytic characteristics when modified with an alkanethiol shell. Therefore, it is not surprising that the number of oxidation events in our experiments also dropped roughly exponentially (β  =  0.29 Å^−1^) with increasing chain length, as presented in Figure 2c. We confirm that oxidative conversion requires the nanoparticles to reach the electrode's cut‐off tunneling distance to enable heterogeneous charge transfer. Thus, the longer the ligand length, the lower the possibility of electron tunneling and the potential at the particle to drive the reaction; see Figure 1a,b. This also implies that thin‐shelled particles might become oxidized at distances slightly further away than thick‐shelled particles.

The impact of the ligand shell on electron tunneling can also be analyzed by examining individual current transients.^[^ ^11^, ^14^, ^43^, ^46^, ^47^ ^]^ To this end, we evaluated for each particle variant, the distributions of maximum current amplitude and impact duration for all recorded current spikes (Figure 2d,e, based on 120 s of recordings from 15 electrodes) and generated histograms of estimated particle sizes (Figure 2f) by integrating the current spikes to calculate the injected charge per collision and converting it to a corresponding particle size, assuming a spherical shape. Monitoring the particle sizes enabled us to distinguish partial from complete oxidation, with the latter indicating that all atoms in a 20 nm‐sized silver particle have been oxidized.

The current amplitudes in Figure 2d for particles with a thin shell (*n* = 3) are spread over a wide range of values with a median of ≈ 165 pA, compared to the substantially smaller amplitudes with a median of ≈ 65 pA for ligand shells *n*  ≥  8. At the same time, the duration of the current peaks increases with the shell thickness for ligands from *n* = 3 to *n* = 8 until it decreases again for particles with the thickest shell (*n* = 11). In summary, a confined particle shell (*n* = 3) supports a fast oxidation process, visible as a short, high‐amplitude transient, whereas larger shells (*n* = 8) lead to smaller current amplitudes and increased durations, reflecting a slower overall process. Our findings match previous work,^[^ ^46^ ^]^ where the oxidation currents at lower electrode potentials, or, in our case, lower effective potentials at the nanoparticle surface φ_NP_, were kinetically limited. In agreement with our data, Ma et al.^[^ ^43^ ^]^ also reported generally higher but more widespread amplitudes for higher overpotentials. Figure 2f shows that the oxidation of particles with thick shells is likely incomplete as the extracted particle sizes significantly deviate from the expected value of 20 nm. In contrast, thin particle shells allow full oxidation because they can access regions of higher potential and enhanced tunneling efficiency during their random walk. We further attribute the deviating results for the particle shell with *n* = 11 to the significantly suppressed electron transfer together with a bias in the analysis procedure^[^ ^47^ ^]^ as extended transients with small amplitudes (e.g., seen in Lu et al.)^[^ ^14^ ^]^ might be masked by the noise floor of ≈25 pA peak‐to‐peak and the ringing‐artifact of the amplifier (see Figure 2a). In addition to the oxidation peaks, we observe for *n* = 11, a few current dips (negative amplitudes range from 10 to 50 pA; see Figure S4, Supporting Information) not present elsewhere in the data. This finding suggests that particles with an extended shell may interact with the electrode capacitively but remain unoxidized—silent—upon their impact with the microelectrode.^[^ ^69^ ^]^

However, the trends seen in Figure 2 might not exclusively stem from the (theoretical) difference in ligand length. Most crucial, the monolayers’ ordering, compactness, and stability are not similar among the ligand ensemble.^[^ ^21^, ^62^, ^70^ ^]^ For instance, mercaptopropionic acid (─COOH, *n* = 3) exists typically in both the *gauche* and *anti* conformation on silver nanoparticles, whereas mercaptoundecanoic acid (─COOH, *n* = 11) presents predominantly, the upright *anti* conformation.^[^ ^71^ ^]^ In general, it has been observed that alkanethiols with shorter chains are more permeable; thus, they experience a faster and stronger degradation, which is also slightly visible in our optical studies in Figures S1 and S3, Supporting Information and may reflect pinhole defects for shorter chains.^[^ ^72^, ^73^, ^74^, ^75^, ^76^ ^]^ As electrochemical methods were shown to be extremely sensitive to surface defects and structural changes in planar alkanethiol monolayers,^[^ ^77^, ^78^ ^]^ our results in Figure 2 might be influenced similarly. However, within the runtime of our 2‐min experiment, we considered the particle variants to be colloidally stable; see Figure S3, Supporting Information.

In addition, adsorbing alkanethiolate ligands alter the surface charge of the interfacial metal atoms; thus, affecting the particle's surface potential/Fermi level, which might impact our results.^[^ ^26^, ^34^, ^59^, ^60^, ^79^, ^80^, ^81^ ^]^ Although all ligands bind via thiol bonds to the particle, variations in the monolayer structure might play a significant role. Research on gold nanoparticles pointed out that the phenomenon is again length‐dependent, with longer ligand chains inducing higher surface potentials.^[^ ^12^, ^32^ ^]^

Another aspect neglected is the ongoing contamination of the electrodes during the experiment, both by released alkanethiol molecules and by forming metal‐oxides due to the high overpotential.^[^ ^82^, ^83^ ^]^ Assuming a thiol footprint of 21.5 Å^2^ and an impact frequency of 1.25 Hz (data for *n* = 3), we might expect ≤ 0.4% of the electrode surface to become covered during a 120 s‐long impact experiment.^[^ ^21^, ^84^, ^85^ ^]^ Although the coverage seems to be low, (spatially distributed) electrode impurities can have severe effects on the oxidation.^[^ ^86^ ^]^ However, we did not observe ligand‐specific differences in the electrode performance during the time course of our experiments.

### 2.3 Oxidation Potential and Redoxactivity

**Figure 3:**
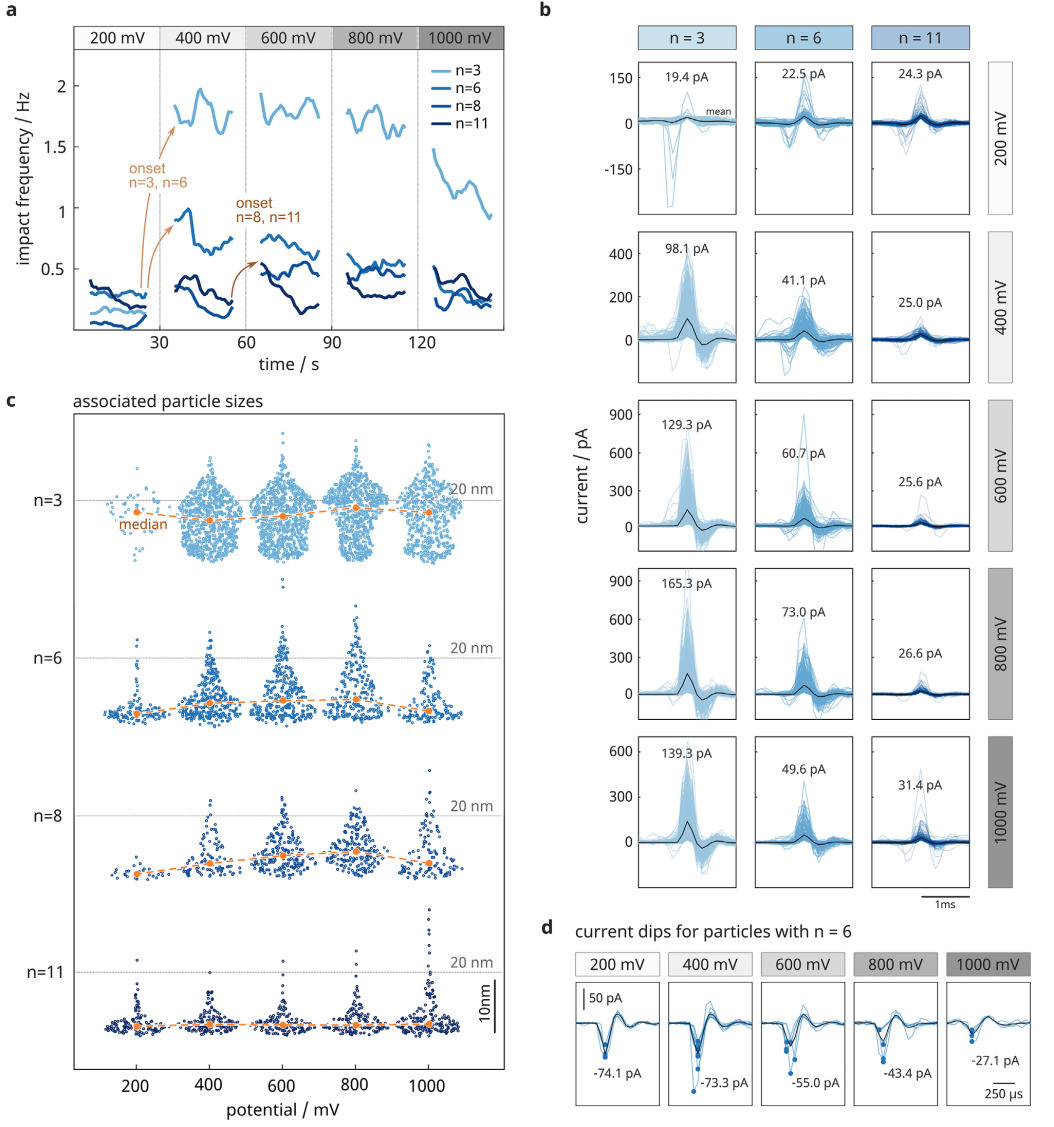
Effect of applied potential on the collision rate. The data were obtained from recordings of 30 pM 20 nm‐sized silver nanoparticles with carboxyl‐terminated alkanethiol coronas of different chain lengths *n* in 30 mM KCl. a) Temporal evolution of the mean impact rate, calculated from parallel recordings of 15 electrodes. b) Offset‐corrected overlays of all identified current peaks within a 2 ms time window. The black line shows the mean transient behavior, and the current value corresponds to the mean current maximum. c) Swarm plots of the estimated particle sizes for different applied potentials. Median values for each size distribution are shown in orange, and the gray line indicates the expected particle size of 20 nm. Additional statistical data can be found in Figure S5, Supporting Information. d) Overlay of current peaks with a preceding current dip exceeding |15 pA| for particles with chain length *n* = 6 at various bias potentials. The values represent the averaged negative transient. The mean duration of the preceding current dips ranges between 380 and 450 µs, showing no clear dependence on the potential. The transferred charge scales linearly with the potential (see Figure S7, Supporting Information), yielding a differential capacitance of ≈9 fF. The potential values are reported with respect to Ag/AgCl.

In a subsequent study, we varied the potential at the electrode successively every 30 s and again recorded impacts from 30 pM particles in 30 mM KCl solution as we were further interested whether higher interfacial potentials could facilitate more (complete) oxidations. Depending on the shell size, we expect to observe a notable transition with increasing applied potential because once the potential in solution is sufficient to promote electron transfer efficiently, the colliding or silent (sitting inactively at the electrode) particles should be converted. For instance, Krause et al.^[^ ^47^ ^]^ reported such a transition for a bias potential ≥ 300 mV in electrolytes with Cl^−^ below 50 mM. To robustly detect the transition behavior, we determined an instantaneous impact rate using the collision event list from 15 channels and applied a 1‐s sliding window with a 50% overlap. As shown in **Figure** **3**a, the temporal evolution of the collision frequency during potential stepping aligned well with our expectations. For instance, a potential of 400 mV was sufficient to oxidize particles with short‐chained (*n* = 3, 6) shells, whereas thicker shells (*n* = 8, 11) required potentials ≥ 600 mV. Similar to the results in Figure 2a, we generally observed in Figure 3a, higher impact rates for particles with thinner coronas. However, increasing the bias potential even further to 1000 mV was not beneficial as the impact rates either remained similar to those at lower potentials (*n* = 11) or even decreased (*n* = 3, 6, 8). This can be explained by interfering metal oxidation of the platinum electrode, a stronger adhesion of thiol molecules, or local changes in the electrolyte due to the onset of hydrolysis,^[^ ^25^, ^83^, ^87^ ^]^ as we measured offset currents on the order of 10 nA for potentials ≥ 600 mV. Last, the jump‐decrease behavior of the impact rate after each potential step suggested that there might indeed be silent particles (for *n* ≥ 6) close to the electrode for which the pre‐step potential could not facilitate tunneling. At the same time, increasing the potential also affects mass transport because electrokinetic (electro‐phoretic and ‐osmotic) particle fluxes scale with the electric field in solution, which could promote higher impact rates.^[^ ^83^, ^87^, ^88^, ^89^ ^]^

We typically observe for 200 mV a negative steady‐state faradaic background current of –20 nA and for higher potentials positive currents of 5 to 30 nA. If electrophoretic particle migration was a major contributing factor, we would expect that the impact rate linearly correlates with the faradaic background current (see also Figure 1c), which we did not observe in our data. However, the influence becomes unclear in the case of incomplete particle oxidation as a particle may only partially convert upon the first impact, potentially escape, and then re‐impact again.

In addition, we evaluated the current transientsin Figure 3b (statistical data given in Figure S5, Supporting Information) and computed associated particle sizes in Figure 3c. As expected, we observed higher current amplitudes with higher potentials and shorter chain lengths. However, only for a thin particle shell (*n* = 3) did the potential substantially alter the proportion between partial and complete oxidations. For particles with *n* ≥ 6, increasing the potential enabled more charge to be transferred but still not close to the amount expected for complete oxidation, highlighting the critical role of electron tunneling through the shell. The widespread size distributions in Figure 3c further indicate a variable particle redox activity after modification, which Bard and co‐workers have also observed in the case of platinum nanoparticles.^[^ ^17^ ^]^ Moreover, the data suggest that a higher tunneling barrier (*n*  ≥  6) cannot easily be overcome by higher potentials because associated side effects such as water splitting and the oxidation of the platinum electrode impede the results.^[^ ^86^ ^]^ We investigated the impact of concomitant electrode oxidation and performed similar experiments in untreated and N_2_‐purged electrolyte solutions (see Figure S6a, Supporting Information) and observed severe consequences as the median impact rate was 54% and 43% lower for 800 and 1000 mV, respectively, when dissolved oxygen was present. Moreover, the untreated electrolyte featured mainly small amplitude peaks, as seen in Figure S6b, Supporting Information, suggesting incomplete oxidation events. In summary, we noticed that electrode oxidation strongly interfered with the experimental outcome but we decided to establish the dataset at standard (environmental) conditions to ensure the best comparability to other work.

Interestingly, the overlays of all current transients in Figure 3b reveal a second characteristic feature, a transient current dip preceding the typical oxidation spike, predominantly present for low bias potentials and thicker particle shells. For instance, 3.5% and 5.2% of all oxidation peaks for *n* = 3 and *n* = 11 had a current dip at 200 mV, whereas at 1000 mV, only 0.3% and 1.4% showed this behavior (see also Tables S1–S3, Supporting Information). The amplitude of the current dips is typically on the order of tens of pA but decreases with the potential, as exemplified in Figure 3d for *n* = 6 and varying potentials. We evaluated the area under the curves; see Figure S7b, Supporting Information, and found negative charges steadily decreasing from 14 to 5 fC, a feature well‐known for capacitive interactions.^[^ ^81^, ^90^ ^]^ Capacitive particle impacts have been mainly investigated for larger species but were also recently emphasized for platinum nanoparticles.^[^ ^91^, ^92^, ^93^, ^94^, ^95^, ^96^ ^]^ As the electrical double layer of the particle–electrolyte differs from that of the electrode–electrolyte (as illustrated in Figure 1c,d), the approaching particle causes the charges at the electrode–particle–electrolyte interface to transiently redistribute. Eventually, the particle may (Figure 3d) or may not (current dips in Figure S4, Supporting Information,) oxidatively dissolve; while, the electric contact is established. It is therefore not surprising that we observe the capacitive transients mainly under conditions where the faradaic process is to some degree impeded, for instance, at low bias potentials or thick particle shells. Otherwise, the faradaic process is fast enough and dominates over the capacitive transient. In addition, we find that the oxidative peaks for those without preceding negative transient (for chain length *n* ≤ 8 and at potentials ≥ 400mV) are at least 1.5 and up to 4 times higher than the peaks with the negative transient, which overall reflects the great variability in physicochemical particle characteristics.

### 2.4 Effect of Terminal Group on Particle Redox Activity

**Figure 4:**
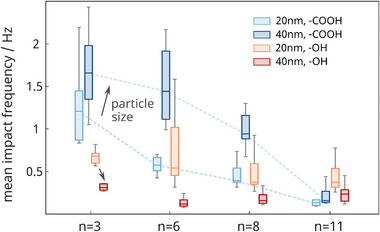
Particle impact frequency as a function of ligand length, terminal group, and particle size. The graph depicts boxplots with median, 25th and 75th percentiles, and minimum and maximum values (error bars) based on recordings from a subset of 15 electrodes. The experiments were conducted with 30 pM particles immersed in 30 mM KCl and a potential of 800 mV versus Ag/AgCl applied for 2 min. Note that the data for 20 nm‐sized COOH‐terminated particles (light blue) stems from the same experiment presented in Figure 2. A 3‐way ANOVA test (see Table S4, Supporting Information) provides statistical significance for all three aspects.

**Figure 5:**
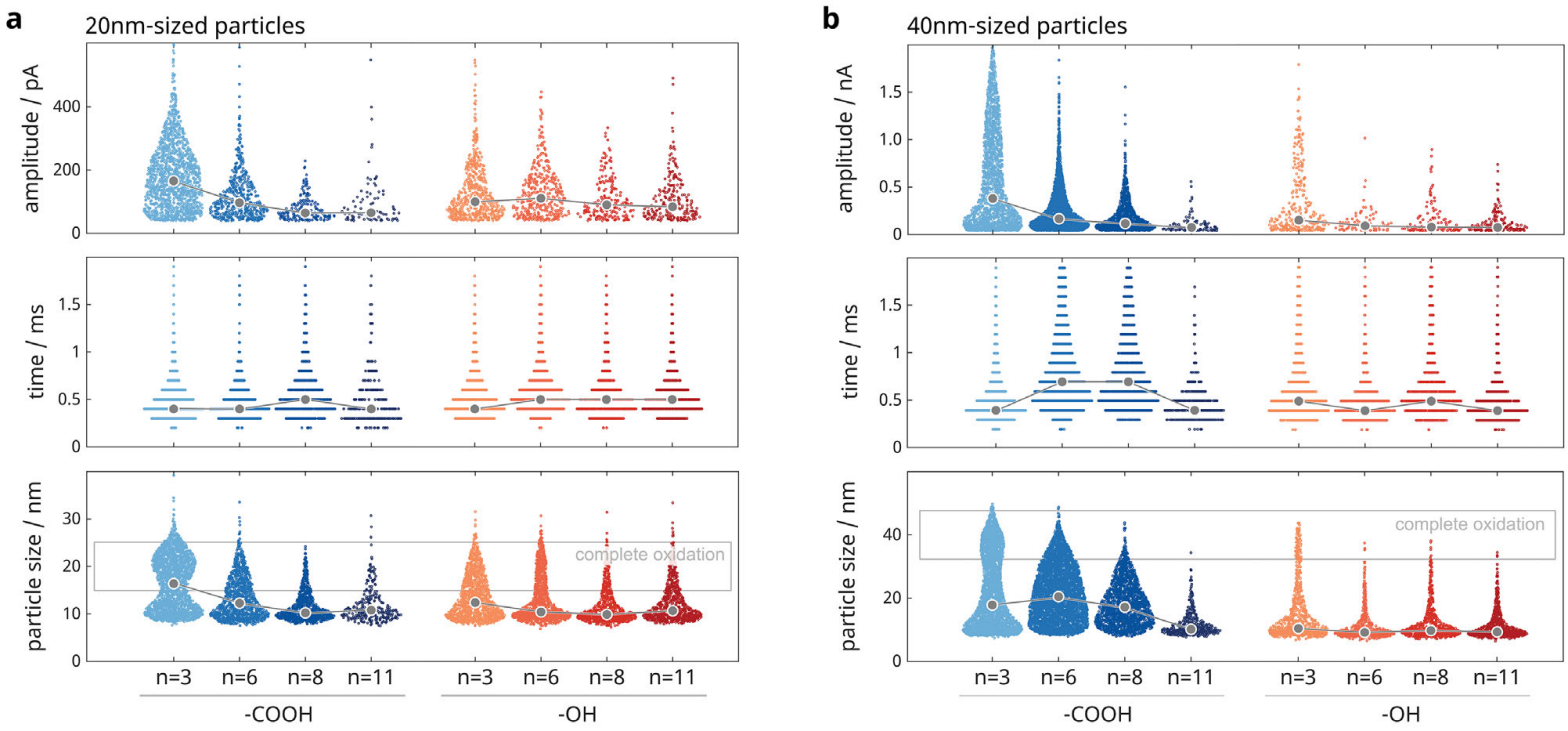
Statistical data of the current peaks, including maximum current amplitudes (top), impact durations (middle), and particle sizes associated with the injected charge upon collision (bottom) for a) 20 nm‐sized and b) 40 nm‐sized nanoparticles. The data is based on 15 individual electrode recordings during a 2 min‐experiment using 30 pM silver nanoparticles, 30 mM KCl, and an applied potential of 800 mV versus Ag/AgCl. Medians are depicted in gray. Preceding current dips for OH‐terminated particles, similar to Figure 3d, are further visualized in Figure S9, Supporting Information. The associated delivered charges per impact are given in Figure S10, Supporting Information.

As recent literature highlights the critical role of the monolayers’ terminal group,^[^ ^15^, ^16^, ^26^ ^]^ we were interested in how silver particles with OH‐terminated shells behave differently than COOH‐terminated ones. We focused on these moieties because they are commonly used to passivate metal surfaces in electrochemical sensing.^[^ ^97^ ^]^ Furthermore, we functionalized particles of two different sizes, 20 and 40 nm, to see if the results were consistent. Overall, we performed impact experiments with 16 different particle variants, where we immersed 30 pM particles in 30 mM KCl solution and biased the electrodes to 800 mV versus Ag/AgCl. **Figure** **4** presents the mean impact rate for all species across a 2‐min experiment. The amplitudes, durations, and associated particle sizes are provided in **Figure** **5**, and exemplary raw current traces are illustrated in Figure S8, Supporting Information. In addition, experimental results for impacts of 20 nm‐sized, OH‐terminated particles during a stepwise potential increase (similar to Figure 3) are given in Figure S9, Supporting Information.

The data in Figure 4 show very different results for the two moieties. Surprisingly, the impact rate of the 20 nm‐sized OH‐terminated particles (orange) varies only slightly with the chain length, which contrasts our previous results for the COOH‐terminated particles (light blue). The same behavior can also be observed for the larger 40 nm‐sized particles (dark blue vs red in Figure 4). This is unexpected as studies on electron transfer across planar OH‐terminated monolayers reported slightly different transfer rates but typically, a similar chain length dependence.^[^ ^21^, ^65^ ^]^

There are two primary explanations for why the behavior could be so different. First, the difference in the impact rate could reflect the structural integrity of the monolayers. It is known that COOH‐terminated monolayers have more defects; and thus, are less stable than OH‐terminated monolayers.^[^ ^98^, ^99^, ^100^ ^]^ The reason is the formation of hydrogen bonds between the terminal COOH‐moieties before the molecules can form closely packed, well‐ordered monolayers. In turn, the monolayer's structure substantially influences the particles’ colloid stability, which we assessed in Figure S3, Supporting Information via UV–vis spectra. Although the data suggests that OH‐terminated 20 nm‐sized particles might be slightly more stable than their COOH counterparts, we did not observe a similar effect for the 40 nm‐particles. Therefore, we infer that the trend depicted in Figure 4 cannot be solely attributed to the presence or absence of pinholes in the particle shells. Instead, it most likely reflects differences in the particles’ surface chemistry.^[^ ^11^, ^26^ ^]^ Recent studies on electrocatalytic and oxidative impacts have reported the critical influence of electrostatic interactions on the adsorption energy and particle residence time.^[^ ^11^, ^15^, ^29^, ^42^, ^101^, ^102^ ^]^ In addition, the terminal group influences both the electron transfer rate by altering the particles' Fermi level and the electron tunneling due to variations in monolayer packing and hydration shells.^[^ ^21^, ^26^, ^103^ ^]^ At moderate pH values of 7.5, the COOH‐moiety is partly deprotonated, leading to a more negatively charged particle shell than the OH‐moiety.^[^ ^104^ ^]^ Hence, we expect a stronger attraction of COOH‐terminated particles to the positively biased electrode. The statistical analysis of the current peaks in Figure 5 supports this hypothesis.

Overall, the COOH‐termini result in slightly shorter current peaks in most cases (e.g., the mean values for ─COOH and ─OH in case of 20 nm, *n* = 3 particles differ by ≈ 50 µs), but significantly higher current amplitudes (e.g., mean values of 166 and 78 pA for ─COOH and ─OH in the case of 40 nm, *n* = 6 particles), which likely indicate differences in the particle trajectories approaching the electrodes.^[^ ^43^ ^]^ As a result, particles with COOH‐termini tend to undergo complete oxidation more readily. Our findings align with those of Dery et al.,^[^ ^16^ ^]^ who also observed more complete oxidations at conditions where particles were electrostatically attracted to and held at the electrode by a positively charged layer. It is, therefore, also not surprising that the impact of the increasing tunneling barrier with chain length was most pronounced for particles with a COOH‐moiety. Krause et al.^[^ ^15^ ^]^ also observed that electrodes functionalized with mercaptoundecanol (*n* = 11, ─OH) experienced more particle collisions compared to those with mercaptoundecanoic acid (*n* = 11, ─COOH). Furthermore, Unwin and co‐workers^[^ ^29^ ^]^ pointed out that the (transient) formation of hydrogen bonds with the electrode could capture particles within the tunneling region, extending collision durations for COOH‐terminated alkanethiol monolayers. Recently, White and co‐workers^[^ ^30^ ^]^ also reported platinum particles to adhere stronger to hydrophilic OH‐terminated alkanethiol monolayers than to hydrophobic ─CH_3_ end groups, where adjacent compact hydration layers could facilitate elastic collisions.

### 2.5 Influence of Particle Size

Given the strong influence of the monolayer's terminal group, we see only a subordinate effect of the particle size: in the case of OH‐terminated particles, the impact rates in Figure 4 decay only slightly with ligand length, but the 40 nm‐sized particles typically show fewer impacts than the 20 nm ones, for example, ≤ 50% for *n* = 3, 6, and 8. Conversely, for COOH‐ligands, it is the opposite effect: the larger particles show more impacts for all ligand lengths than the smaller particles. We attribute this to the COOH‐monolayer's higher permeability and the significant electrostatic attraction caused by deprotonated carboxyl groups.

Moreover, the 40 nm‐sized particles exhibit a larger contact area with the electrode because of their smaller curvature and overall larger size, resulting in decreased electrical resistance.^[^ ^97^ ^]^ Consequently, larger COOH‐terminated particles are expected to benefit more from additional attractive interactions or monolayer defects. In contrast, for OH‐terminated particles, weaker attraction or denser monolayer packing will likely result in incomplete oxidation (as evident in Figure 5b) and multipeak behavior.^[^ ^105^ ^]^ Unfortunately, we cannot directly probe the larger particles' multipeak characteristics as the bandwidth of our amplifier system limits us. With our setup, small amplitude currents are likely a smeared representation of short amplitude bursts with a subsequent escape of the particle, resulting in partial oxidations.^[^ ^105^, ^106^, ^107^ ^]^ Especially, the observed longer current durations (reflecting the residence times) support the electrostatic attraction for ─COOH, whereas the higher amplitude suggests more permeable monolayers because the current amplitudes can be correlated with the particle distance to the electrode (e.g., via Butler–Volmer and Poisson–Boltzmann). Another reason for the increased impacts of larger particles can be that the contribution to the energy barrier from solvent reorganization at the outer sphere is higher for smaller particles because more atoms are superficial; this renders electron transfer less effective for smaller particles.^[^ ^63^ ^]^ However, as predicted by Plieth and Henglein,^[^ ^108^, ^109^ ^]^ the particle's Fermi level (thus, its redox activity) is supposed to shift to lower values as the particle size decreases. Zamborini and co‐workers^[^ ^110^ ^]^ experimentally verified this hypothesis via linear sweep voltammetry of immobilized nanoparticles on glass/ITO electrodes. In their case, the particles were already coated onto the electrodes, which differs from our experimental setup, where the particle trajectories close to the electrode were an essential aspect.

So far, we have exclusively considered the dynamic interactions of the particles with the electrode, but the collision rate likewise depends on the flux of particles drawn from the bulk. All our experiments are operated under mass‐transport limitations. Nevertheless, impact recordings are reported to be still extremely sensitive to changes in the subsequent reaction dynamics.^[^ ^9^, ^25^, ^43^, ^45^, ^111^ ^]^ In first approximation, particle transport is mainly driven by diffusive motion and electrophoretic migration.^[^ ^88^, ^111^, ^112^ ^]^ The particles’ diffusion constant *D* scales with 1/*r* (according to the Stokes–Einstein relation), and consequently, the particle collision also scales with 1/*r* as the steady‐state particle flux depends linearly on *D* (based on the Saito equation). Therefore, by neglecting size‐dependent boundary effects,^[^ ^113^ ^]^ we expect for 40 nm‐particles, a collision rate reduced by a factor of two compared to 20 nm‐particles. The electrophoretic particle flux stays independent of the particle size for a sufficiently high concentration of supporting ions (Smoluchowski approximation of a thin Debye length; see also Figure 1d). It depends on the surface charge density and the electric field.^[^ ^88^ ^]^ Thus, ligand shells with varying charges should theoretically produce different particle fluxes, provided all other factors remain constant. Although we measure slightly higher negative zeta‐potentials for 40 nm particles (but generally zeta‐potentials between –31 and –43 mV for all variants; see Figure S1d, Supporting Information), the minor differences cannot explain the drastic deviations in impact frequency regarding size and end group. Clearly, our predictions for the impact rate based on bulk mass transport contradict the data in Figure 4, which again highlights the critical role of surface chemistry and the dynamic interactions at the interface. In fact, the slower diffusive motion of 40 nm particles, even pronounced near the electrode surface,^[^ ^113^ ^]^ can also be beneficial because the random walk inside the tunneling region is slowed down, lowering the likelihood of particles escaping.

### 2.6 Influence of Electrolyte Composition

**Figure 6:**
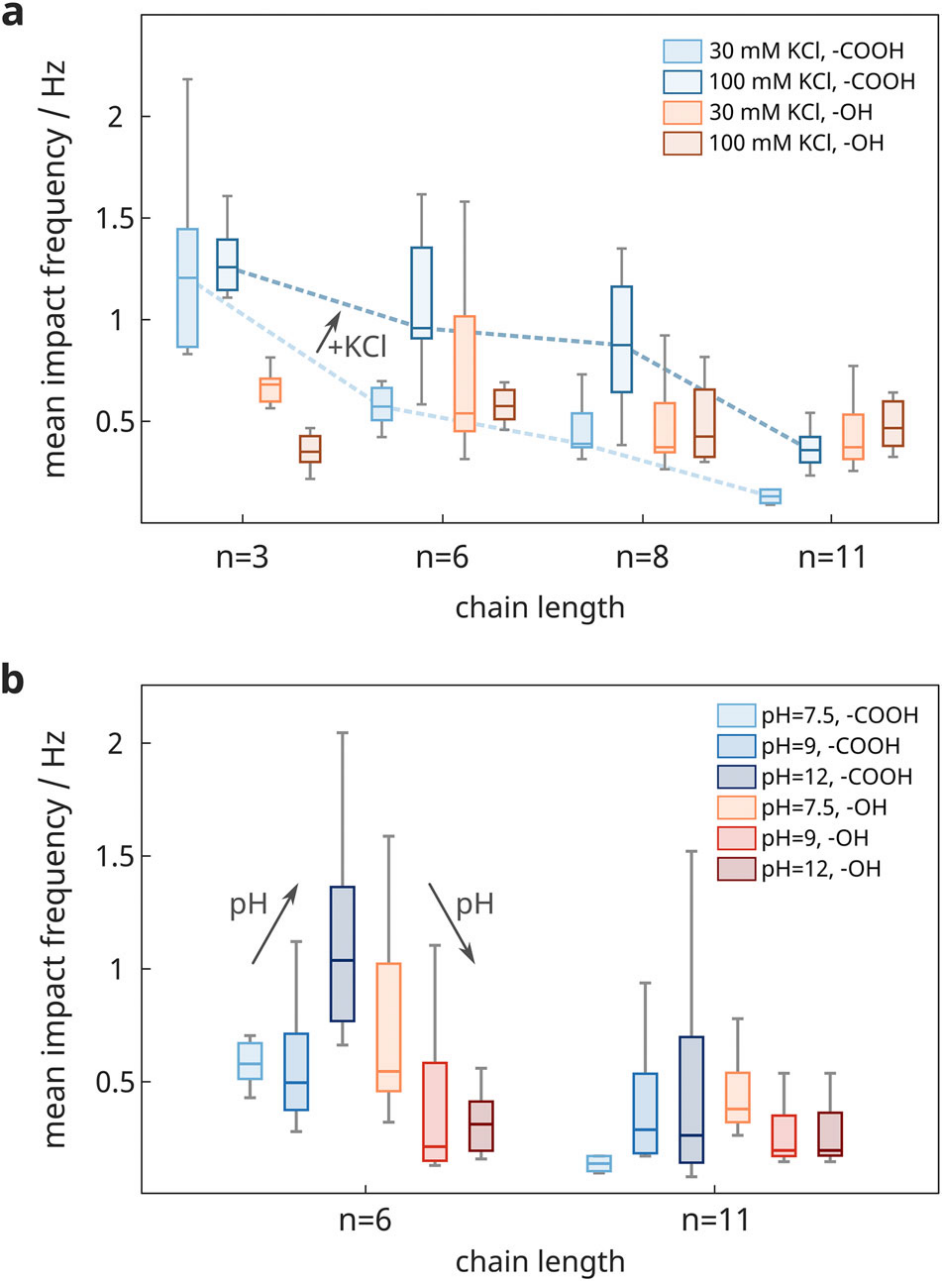
Critical interplay of electrolyte composition and particle corona. a) Influence of the electrolyte concentration on the mean impact rate of 20 nm‐sized particles. b) Mean impact rates of 20 nm‐sized particles with chain length *n* = 6 and *n* = 11 measured in 30 mM KCl at different pH values. The pH was adjusted via KOH. In both experiments, the applied oxidation potential was set for 120 s to 800 mV versus Ag/AgCl, and 30 pM particles were suspended. Notably, the experimental data for particles recorded in 30 mM KCl (at pH 7.5) is identical to that presented in Figure 4.

The discussion above identifies the particles’ surface chemistry as crucial, which is generally not only governed by the ligand type but also by the surrounding electrolyte.^[^ ^25^, ^26^, ^47^ ^]^ Typical impact experiments with silver nanoparticles are conducted at low salt conditions, for instance, at 30 mM KCl, where the stability of (citrate‐capped) particles is preserved.^[^ ^114^ ^]^ At the same time, variations in the chloride concentrations ≤ 50 mM are shown to affect the impact frequency, current amplitudes, and durations.^[^ ^44^, ^46^, ^47^ ^]^ For overpotentials ≥ 200 mV, the oxidation process becomes dominated by the diffusion of the co‐reactants participating during the dissolution.^[^ ^25^, ^44^ ^]^ Thus, our entire study with 800 mV versus Ag/AgCl and in 30 mM KCl operates in the dissolution‐limited regime to avoid any ambiguities from colloidal instability. To qualitatively explore how results may change, we perform experiments with selected particle variants measured i) in 100 mM KCl (see **Figure** **6**a), ii) in 30 mM KCl solution with increasing pH values (see Figure 6b; Figure S12, Supporting Information), iii) as well as in phosphate buffer saline representing a complex electrolyte with biological relevance (see Figure S13, Supporting Information).

Figure 6a shows that higher co‐reactant concentrations enhance the particles’ redox activity, particularly for ─COOH‐terminated ligands with lengths of *n* = 6 and *n* = 8, highlighting the impact of the double layer extent and dissolution step. Increasing the KCl concentration from 30 to 100 mM reduces the Debye length κ^−1^ from 1.76 to 0.96 nm, effectively doubling the electric field strength at the interface. However, while strong electric fields and rapid dissolution provide optimal conversion conditions,^[^ ^45^, ^47^, ^58^ ^]^ the final outcome ultimately depends on a terminal group that facilitates particle adhesion, as seen for ─COOH particles. In contrast, increasing the ionic strength provides little to no benefit when the particle–electrode interaction is weak, as observed for the ─OH terminal. Moreover, elevated salt concentrations can lead to colloidal instability, which is also slightly evident for the 20 nm particles with *n* = 3, ─COOH functionalization (see Figure S11, Supporting Information).

The predominant role of the terminal group is also apparent in Figure 6b, which shows the redox activity as a function of the electrolyte's pH value. At higher pH values, we observe for ─COOH increased impact frequencies, consistent with the findings of Ma et al.^[^ ^11^ ^]^ and Xu et al.^[^ ^108^ ^]^ This can be explained by a stronger deprotonation of the carboxyl‐group at higher pH values, which also aligns with our findings of substantially longer peak durations (shown in Figure S12, Supporting Information). However, for OH‐terminated particles, the trend in impact rate is vice versa, this time without a clear dependence on the impact duration. As alkaline solutions typically increase particle adsorption to the electrode and simultaneously promote their efficient dissolution also in the presence of citrate as capping agent,^[^ ^11^ ^]^ we conclude that the decrease in collision rate for OH‐terminated particles has to originate from changes at the ligand–electrolyte interface, for instance, structural reorganizations of the shell and adlayers (see also Figure 1d), that potentially affect charge transfer, colloid stability, and adsorption to the electrode. Similarly, the experiments in phosphate‐buffered saline (in Figure S13, Supporting Information), where the OH‐terminated particles exhibit now similar impact rates as the COOH‐terminated ones, demonstrate that nano‐impact studies are affected by complex interconnections of various mechanisms, which are typically delicate to disentangle. Our data suggests that the experimental findings presented here might not necessarily be valid for other combinations of ligand types and electrolytes. At the same time, it becomes apparent that impact‐based sensing applications require a more profound understanding thereof to engineer and tune particle–electrode interfaces on demand.^[^ ^7^, ^9^ ^]^

## 3 Conclusion

In this work, we systematically studied how changes in the particle corona affect the redox activity of silver nanoparticles in stochastic impact experiments. We varied the chain lengths and functional groups of the coatings, evaluated the effect of the applied potential, and assessed how results changed for different particle sizes. The key findings include:

- The terminal group of the particle shell rules the interfacial dynamics. Electrostatic interactions play a crucial role in facilitating efficient charge transfer.
- The effect of the terminal group scales with the particle size as the area of electrical contact is larger. Thus, electrostatic effects are stronger for 40 nm‐sized particles.
- The thickness of the shell is essential as chain lengths *n* > 6 impede charge transfer drastically. The contribution; however, is modulated by the electrostatic interactions and particle size. Thus, we see the strongest effect for 40 nm particles with carboxyl moiety.
- Increasing the bias potential of the electrode (beyond 400 mV) has only a subordinate effect as it can only substantially alter redox characteristics for particles with a very thin shell (chain length *n* = 3).
- Given that colloid stability is preserved, increased ionic strength enhances the particle redox activity, presumably by creating strong electric fields within the double layer. Moreover, higher anion concentrations enhance the particle redox activity by accelerating the dissolution step. However, this effect is only observed for particle shell–electrolyte combinations that ensure sufficient particle adsorption onto the electrode, the most critical substep.
- The electrolyte composition, in terms of pH and ion type, has a crucial role in redox processes at the nanoscale. Our exemplary findings highlight the need for further fundamental research in this regard.

We complement existing research on nanoimpacts recorded from functionalized electrodes by measuring the stochastic collisions of functionalized particles. This approach not only advances the understanding of rationally designing nanoparticle labels for the efficient detection of very diluted species but also underscores the versatility of stochastic impact‐based sensing as a powerful framework for bioassays, capable of detecting modulations in particle behavior with potentially exceptional sensitivity.

The digital method is adaptable to diverse assay architectures. For instance, target analytes may trigger particle release, aggregation, or de‐aggregation, leading to significant changes in collision rates and detected particle sizes.^[^ ^9^, ^115^, ^116^, ^117^ ^]^ Alternatively, analytes may interfere with the particle corona by inducing conformational changes or displacing ligands, thereby altering the redox activity.^[^ ^118^ ^]^ To ensure optimal performance in such assays, the particle shell should be engineered to maximize the difference in signal output between the target's presence and absence. Nanoparticles can also serve as redox‐active labels in classical competitive or sandwich assays, where their interaction with the target or receptor creates local concentration sinks/sources instead of directly modulating redox activity.^[^ ^7^ ^]^ In such cases, balancing the particles' responsiveness to biological input with their redox activity is crucial. As illustrated by a model system in Figure S14, Supporting Information, utilizing mercaptopropionic acid (*n* = 3, ─COOH) and a biotinylated alkanethiol chain, the co‐assembly with non‐specific molecules, helps to preserve higher redox activity compared to assemblies composed exclusively of specific molecules. Overall, our findings highlight that particle adsorption can be fine‐tuned either by selecting appropriate functional end groups or non‐specific spacer molecules or by adjusting the composition of the detection buffer. Modifying the buffer is experimentally more straightforward; however, sensing applications often impose constraints on buffer choice, such as specific pH or the presence of certain ions, which may not align with the requirements for optimal electrochemical detection.

In summary, we believe that, beyond an exclusive emphasis on specific molecules in biosensing, substantial potential lies in the deliberate selection of non‐specific molecules that compose the particle shell. These non‐specific components can profoundly influence sensor performance, particularly by impacting the particles’ detectability in different assays. In addition, our findings highlight the role of detection buffer optimization, which holds the potential to expand opportunities beyond the constraints of biological sensing requirements. Altogether, these perspectives encourage rethinking ligand and electrolyte choices to explore versatile strategies for optimizing particle‐based electrochemical (bio)sensing.

## 4 Experimental Section

### Particle Modification and Characterization

Detailed information on the particle modification and results from optical characterization studies are provided in the Supporting Information and illustrated in Figures S1 and S2, Supporting Information. After confirming the integrity of the particles, their colloidal stability in the electrolytes was assessed via absorbance measurements. Exemplary results for 30 mM KCl are provided in Figure S3, Supporting Information.

### Stochastic Impact Recordings

The stochastic impact experiments were performed with a microelectrode array chip described in previous work.^[^ ^82^ ^]^ The electrode array comprised 62 Pt disk electrodes with a diameter of 8 µm surrounded by a SiO_2_ passivation layer. The amperometric recordings were carried out with a custom‐built 64‐channel amplifier system at a sampling rate of 10 kHz per channel (and a bandwidth of 3.4 kHz) in a two‐electrode configuration using an Ag/AgCl reference electrode (3 mM NaCl gel electrode, BASi). Throughout the manuscript, all potential values are reported with respect to the Ag/AgCl reference electrode.

The chip was cleaned and activated between subsequent experiments according to the protocol of earlier work.^[^ ^7^ ^]^ Here, a VSP‐300 potentiostat (BioLogic Instruments, France) was used in a three‐electrode configuration, consisting of a coiled‐platinum wire as counter electrode and a Ag/AgCl electrode as a reference, respectively. Prior to each experiment, the electrolyte solution was freshly spiked with nanoparticles in a separate tube to achieve homogeneous mixing and mitigate interfering particle adsorption. After immersing the electrode array, the oxidation potential—either a constant voltage of 800 mV or consecutive voltage steps of 200 mV ranging from 0 mV to 1 V—was applied, and impacts were recorded. The experiment conducted at a constant potential had a duration of 120 s, whereas the experiment with increasing voltages extended over 150 s.

The current traces were analyzed using a custom algorithm in Matlab.^[^ ^82^ ^]^ In short, detrending of the current traces followed by thresholding at 15 pA was used to extract the current transients associated with the particle collisions. Integration of the current transients over time yielded the charge injected upon impact, which could be converted to a particle size distribution—under the assumption of complete oxidation and a spherical geometry—via:

(5) $$d_{p}=2\cdot \sqrt[{3}]{\frac{3M_{\mathrm{Ag}}Q}{4\pi zF\rho_{\mathrm{Ag}}}}$$

where *d*_p_ is the diameter of the nanoparticle, *Q* is the delivered charge, and *F* is the Faraday's constant. Moreover, *M*_Ag_, ρ_Ag_, and *z* are the molar mass, the mass density, and the valency of Ag, respectively. In the Results section, associated particle diameters *d*_p_ are reported instead of delivered charge *Q* to highlight deviations of the experimental data from the expected particle sizes of 20 and 40 nm. The mean impact rates were computed for all working channels (well‐connected and non‐noisy electrodes based on their current noise in control recordings), and a subset of 15 electrodes showing the highest impact rates was used to compute the data reported here. This approach accounts for differences across chips, as well as for differences within one chip.

## Conflict of Interest

The authors declare no conflict of interest.

## Supporting information



Supporting Information

## Data Availability

The data that support the findings of this study are available from the corresponding author upon reasonable request.
