# Supplementary material for: Stochastic Impact Electrochemistry of Alkanethiolate‐Functionalized Silver Nanoparticles
Source: Small. 2025 Mar 13;21(16):2410306. doi: 10.1002/smll.202410306 (PMC12019921; doi:10.1002/smll.202410306)
Supplement: Supplementary file 1 — Supporting Information [file SMLL-21-2410306-s001.docx]

Supporting Information

Stochastic Impact Electrochemistry of Alkanethiolate-Functionalized Silver Nanoparticles

*Lennart J. K. Weiß*, Marta Nikić, Friedrich C. Simmel, Bernhard Wolfrum*

# Experimental Section for Nanoparticle Preparation

### Materials

The nanoparticles (20 nm and 40 nm diameter, citrate-capped, 0.02 mg/mL in aqueous solution) and all alkanethiolates (2-mercaptoethanol, 6-mercapto-1-hexanol, 8-mercapto-1-octanol, 11-mercapto-1-undecanol, 3-mercaptopropionic acid, 6-mercaptohexanoic acid, 8-mercaptooctanoic acid and 11-mercaptoundecanoic acid) were purchased from Merck, Germany. Modified phosphate-buffered saline (PBS) solution, potassium chloride (KCl), potassium hydroxide (KOH), sulfuric acid (H2SO4), sodium citrate, 4-(2-hydroxyethyl)piperazine-1-ethanesulfonic acid (HEPES), ethanol and Tween 20 were also bought from Merck, Germany. Ammonium hydroxide (NH4OH, 28%) was obtained from VWR Chemicals, France. All solutions were prepared via deionized water (conductivity 0.054 μS/cm) from a Berry Pure purification system (Berrytec, Harthausen, Germany). In the case of long-chained alkanethiolates (n ≥ 8), the initial 1 mM stock solutions were prepared with a 1:1 water/ethanol mixture to ensure solubility.

### Protocol of pH-Mediated Nanoparticle Functionalization

The pH-assisted functionalization procedure was adapted from Zhang et al.^[1,2]^ For the study, we used eight different alkanethiolates, with the general structure given by HS-(CH_2_)_n_-COOH/OH and n = 3, 6, 8, and 11. The ligand exchange was performed as follows: 600 µL of 0.02 mg/mL citrate-stabilized nanoparticle suspension was thoroughly mixed with 50 µL of 1% Tween 20 solution in a 1.5 mL tube. Then, 6 µL of alkanethiolate solution (10µM for mercaptoethanol and -hexanol, 100µM otherwise) were added followed by 2 µL of 500 mM sodium citrate at pH 3 to induce the pH-assisted ligand exchange. The mixture was shaken for 15 min at 350 rpm at room temperature until the pH value was readjusted using 6 µL of 500 mM HEPES. Last, additional 2 µL of the respective alkanethiolate solution were added as backfill. The mixture was kept in the fridge at 4°C for at least 12 h prior to the centrifugation / washing step. Note that the conjugation protocol for the 20 nm-sized and the 40 nm-sized particles was the same, as both stock colloids vary only by a factor of 0.5 in their expected total surface area. Prior to centrifugation, 900 uL of deionized water were added to the particle suspensions. In case of 20 nm particles, the suspension was centrifuged once at 10,000 × g and twice at 8,000 × g for 35 min using a MiniSpin plus (Eppendorf, Germany). Similarly, the 40 nm-sized particles were centrifuged at first 8,000 × g and two times at 6,000 × g, each for 20 min. In between, 900 µl of supernatant were replaced by deionized water.

### Particle Characterization and Stability Assessment

After modification, the particle suspensions were characterized via UV/Vis recordings, dynamic light scattering (DLS), and $\zeta$-potential measurements in deionized water. The UV/Vis spectra were acquired with a Specord 210 spectrometer (Analytik Jena, Germany). The DLS and ζ-potential measurements were carried out with a ZetaSizer Nano ZS (Malvern Panalalytical, UK). For ζ-potential recordings, the particle suspensions were purified via Zeba Spin desalting columns (7 kDa MWCO, Thermo Fisher) prior to the measurement. The concentrations of the final particle suspensions were determined by comparing their UV/Vis spectra with the commercially available stock suspensions. Furthermore, the colloid stability in different electrolytes was assessed via successive recordings, e.g. ranging from 0 to 15 min after insertion, to track changes in the spectra that can be associated with dynamic changes in the colloid, such as aggregation or dissolution. The particle morphology and size distribution of the particles were also confirmed by transmission electron microscopy (TEM). Here, 4µl of the as-prepared samples were plated on carbon-coated copper grids (FCF400-CU-50, Electron Microscopy Sciences, Germany) and dried for 20min to 1h. The images were collected using a Philips CM100 at 100 kV.

# Optical Characterization of Nanoparticles Modified with Alkanethiol Ligands

We modified 20 nm- and 40 nm-sized silver nanoparticles with alkanethiol ligands of various chain lengths (n = 3, 6, 8, 11) and end groups (-COOH / -OH), yielding in total 16 different particle species as shown Figure 1a. Our one-pot modification procedure is adapted from Zhang et al. and uses a fast, pH-induced ligand exchange.^[1,3,4]^ We confirmed the successful modification by assessing various physicochemical bulk characteristics of the nanoparticles, such as their localized surface plasmon resonance (LSPR), hydrodynamic diameters, and $\zeta$-potentials (see Figure S1b to d). For instance, the UV/Vis spectra of 20 nm-sized particles in Figure S1b.1 and the LSPR peaks for the complete dataset in Figure S1b.2 depict (in all but one case; 40nm -COOH) a redshift in the spectra, which can be attributed to an increase the local dielectric permittivity stemming from changes in the particle corona.^[5–8]^ In line with literature, we see the LSPR maxima increasing with the chain length for carboxylated ligands.^[6,7,9]^ Surprisingly, the hydroxylated ligands do not behave similarly, as the LSPR peaks are roughly constant per particle size. A possible reason for this could be that hydroxyl-terminated coronas are more affected by residual Tween 20 (from the modification process), probably forming an additional surrounding shell that leads to the constant LSPR. Yet, acidic conditions also facilitate the (partial) oxidation of the outermost Ag atoms of the particles, which might cause a similar red shift in the spectrum.^[10]^ In this case, we would expect the particles to be redox inactive, thus *silent*, in the subsequent electrochemical experiments, but our electrochemical recordings did not support this hypothesis.

We further observe converging LSPR values for both moieties at higher chain lengths. This is reasonable, as the influence of the terminal group on the probed dielectric permittivity diminishes with increasing chain length. Therefore, we attribute the differences for short-chained ligands (n ≤ 6) to structural differences in the monolayers and/or the adjacent hydration shells of the particles. The assembly onto planar surfaces has been studied extensively. It is well-known that longer alkanethiols (n > 10) form more compact and stable layers due to increased van der Waals interactions for the extended alkyl chains.^[11–16]^ In contrast, short ligands are structurally less ordered and more prone to surface defects. Unsurprisingly, the assembly onto highly curved surfaces – nanoparticles – that expose different crystal facets is even less orderly and compact, primarily influenced by structural defects.^[11,17]^


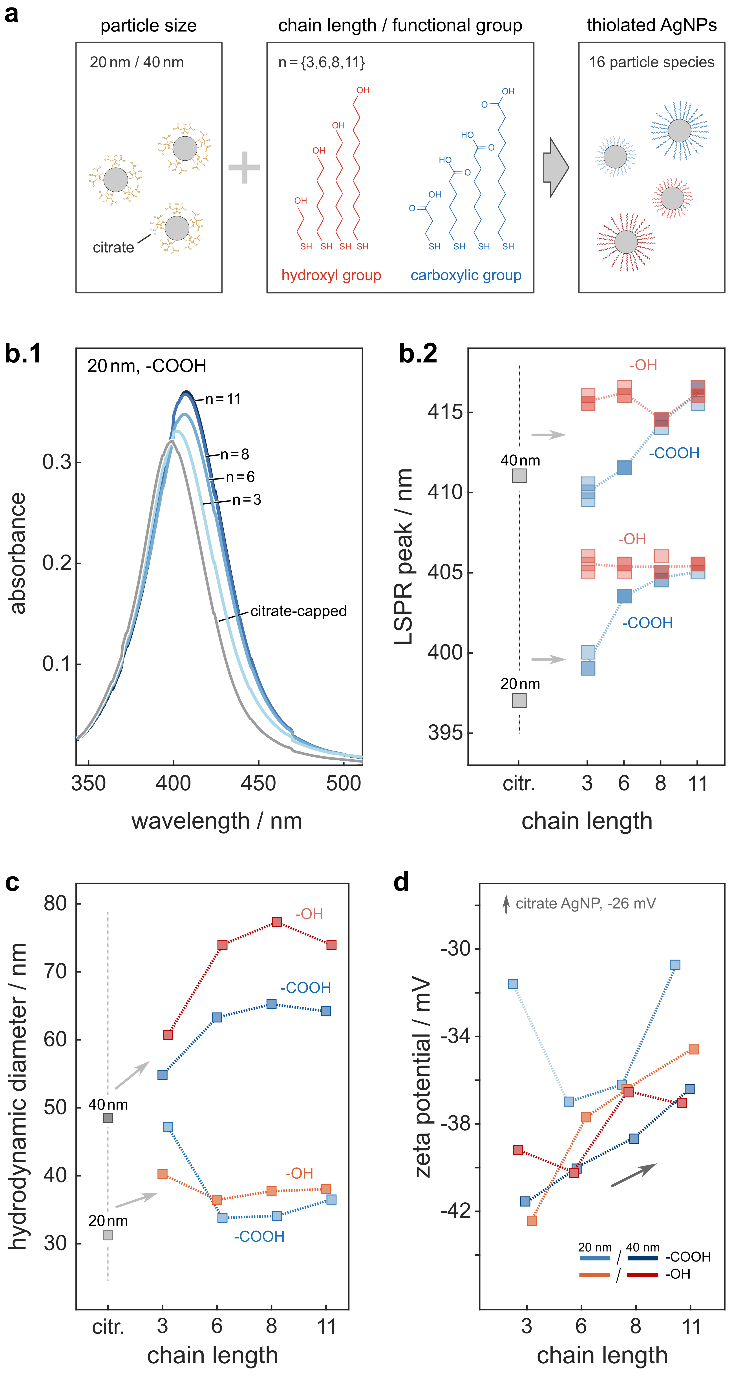


**Figure S1** Optical Bulk Characteristics of modified nanoparticles. (a) Silver nanoparticles with 20 nm and 40 nm diameter were modified with n-alkanethiolates of various chain lengths (n = 3, 6, 8, 11) and terminal groups (-COOH / -OH) (b.1) Exemplary UV/Vis spectra of 20 nm-sized particles with carboxylated ligands indicate a LSPR shift. (b.2) LSPR peaks for all functionalized particles. Each square represents a modification trial. (c) Average hydrodynamic diameters and (d) average $\zeta$-potentials of the modified particles. The data was acquired in deionized water after ligand exchange.

Overall, Figure S1b.2 suggests a robust protocol, as the results from three independent modifications are consistent for all species. However, the adsorption dynamics and the final coverage of the particles are expected to differ across the ensemble, as the chain length, the terminal group, and the curvature were shown to significantly affect the assembly.^[18–23]^ For instance, the outlier in Figure S1b.2 – the blueshift for 40nm-particles, n=3, -COOH, typically associated with leaking Ag^+^ ions – could indicate a decreasing particle size during modification until a stable monolayer has been formed, although TEM images do not show significant changes in the particle size, see Figure S2c.

Second, we measured the hydrodynamic diameters of the particles, see Figure S1c. This value represents an effective particle size governing the diffusive motion. It is typically larger than the sum of the plain particle and the thickness of the coating because the particle carries a hydration shell (see, e.g., the values for citrate). The hydrodynamic diameters increased for all modified species, and for all but one condition (n=3, 20 nm-sized particles), the hydroxyl moiety leads to a larger diameter than the carboxyl moiety. For 20 nm particles with chain length n≥6, the hydration shell of the modified particles is in a similar range than the citrate-stabilized, whereas for 40nm particles, it is not. Our data might be explained by varying hydration depending on the curvature and terminal groups.^[24,25]^ Nevertheless, the recordings are very sensitive to differences in the viscosities of the different samples (from residual Tween 20),^[26]^ but could also reflect the degree of polydispersity in the colloid in conjunction with a recording bias towards larger particles.^[27,28]^

Third, we determined $\zeta$-potentials (depicted in Figure S1d), where we obtained a substantial decrease for all modified (ranging from -31mV to -43mV) compared to the initially citrate-capped particles ($\zeta=$ ‑26mV). As a rule of thumb, particle suspensions are expected to be electrostatically stable for $|\zeta|$>30mV. We see short ligands leading to higher $\zeta$-potentials, which can be explained by an increased surface charge density for the thinner particle coronas. In contrast, the different terminal groups seem to have no (measurable) influence on the $\zeta$-potential, which is to some extent surprising as hydroxyl and carboxyl groups have different surface charges and hydration shells but might be also attributed to interfering physisorbed Tween 20.

Last, we qualitatively assessed the particles’ integrity after modification via TEM imaging. Exemplary data is provided in Figure S2, suggesting that the protocol does not lead to substantial aggregation or shrinking of the particles. Especially in the case of the 40nm, n=3, -COOH particles, we did not observe a clear difference to other particles which is also consistent with the DLS and $\zeta$-potential data in Figure 1c and d.

**
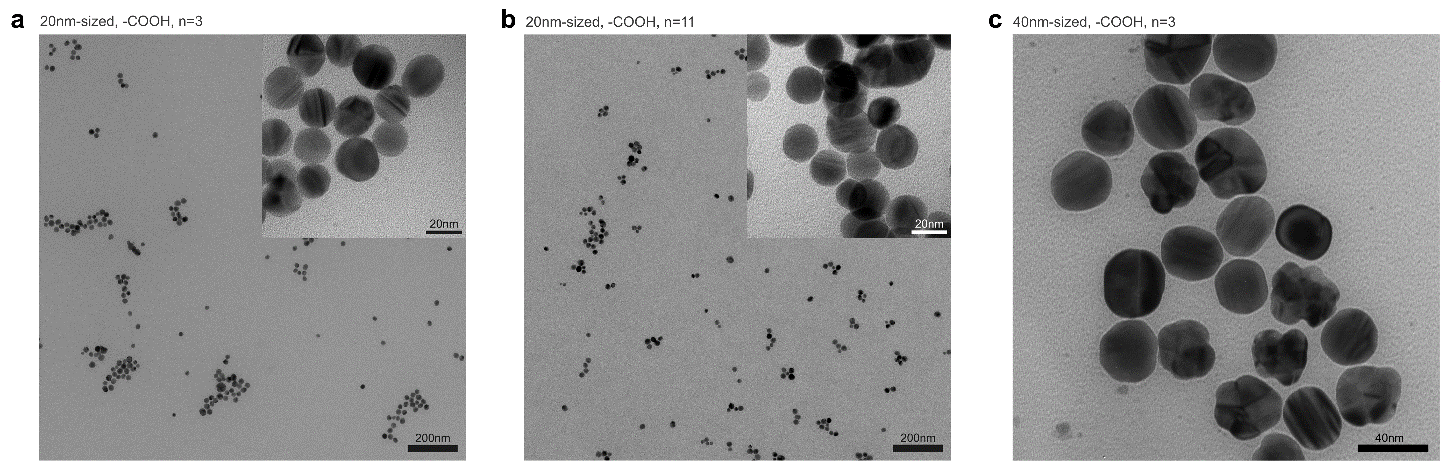
Figure S2** Exemplary TEM images of modified nanoparticles. (a) 20 nm-sized silver nanoparticles after modification with mercaptoproprionic acid (-COOH, n=3) and (b) after modification with mercaptoundecanoic acid (-COOH, n=11). Scale bars 200 nm and in the zoom 20 nm. (c) 40nm-sized particles with a mercaptoproprionic acid (-COOH, n=3) corona. Scale bar 40 nm.

# Colloid Stability of the Modified Silver Nanoparticles

Prior to the impact study, we tested the colloidal stability of the 16 different particle species in 30mM KCl – a typical electrolyte solution in single-impact experiments – via successive UV/Vis recordings, see Figure S3. Based on minor changes (less than a 5% drop in amplitude and absence of secondary resonance modes) in the LSPR spectra, we considered the particles to be stable within the timeframe of an impact experiment. In contrast to all other species, the 20 nm-sized mercaptopropionic acid-capped (-COOH, n=3) particles showed persistently decreasing amplitudes, implying a more fragile corona. In agreement with literature,^[22,29–33]^ we noticed the long-chained ligands to form more stable monolayers, as they are less prone to defects and typically more ordered, thus, more densely packed than short ligands. Furthermore, the spectra emphasize that the corona of 40 nm particles is more stable than that of 20 nm ones, reflecting more ordered assemblies and a higher surface charge density because of the smaller curvature for the larger particles. Last, we observed fewer spectral changes for OH-terminated species compared to particles with a bulkier -COOH moiety. This observation might be attributed to an increased disorder and less compact packing for carboxyl-termini, although stabilizing hydrogen bonds could be formed.^[34,35]^


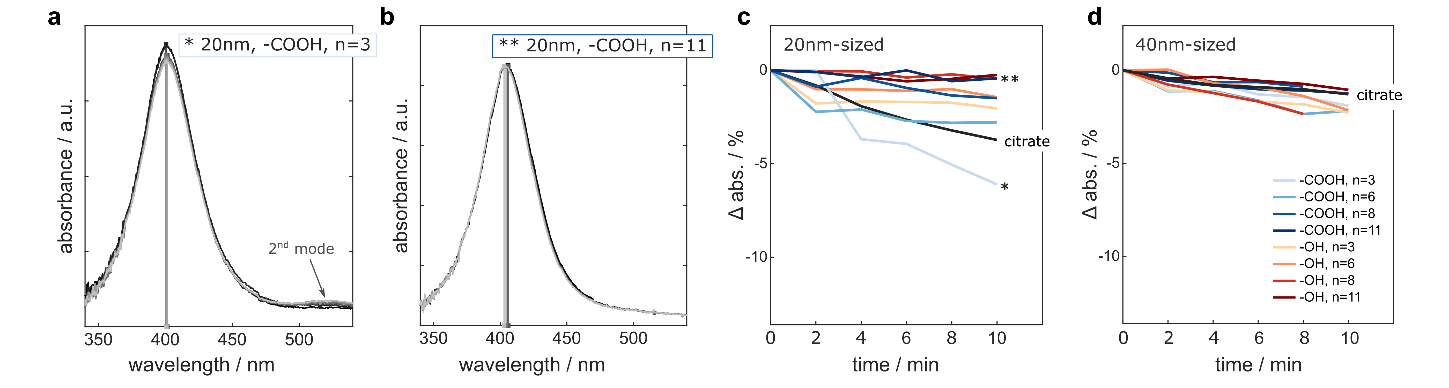


**Figure S3** Colloidal stability of modified nanoparticles in 30mM KCl solution. (a) Exemplary successive UV/Vis recordings of 20 nm-sized silver nanoparticles with a mercaptopropionic acid corona (-COOH, n=3) and(b) with a mercaptoundecanoic acid corona (-COOH, n=11) within the timeframe of 10 min. (c) Relative change in the LSPR peak for all 20 nm-sized modified particles and (d) for all 40 nm-sized particles.

# Current Dips in Impact Recordings


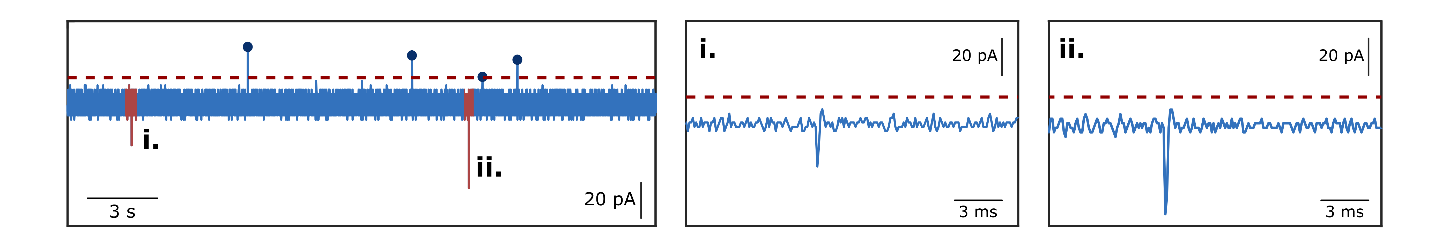


**Figure S4** Exemplary current dips without following oxidation peak in case of 20 nm-sized particles with mercaptoundecanoic acid (-COOH, n=11) corona. The amplitudes typically range between 10 pA and 50 pA.

# Statistical Analysis of Current Peaks for Experiment with Increasing Applied Potential


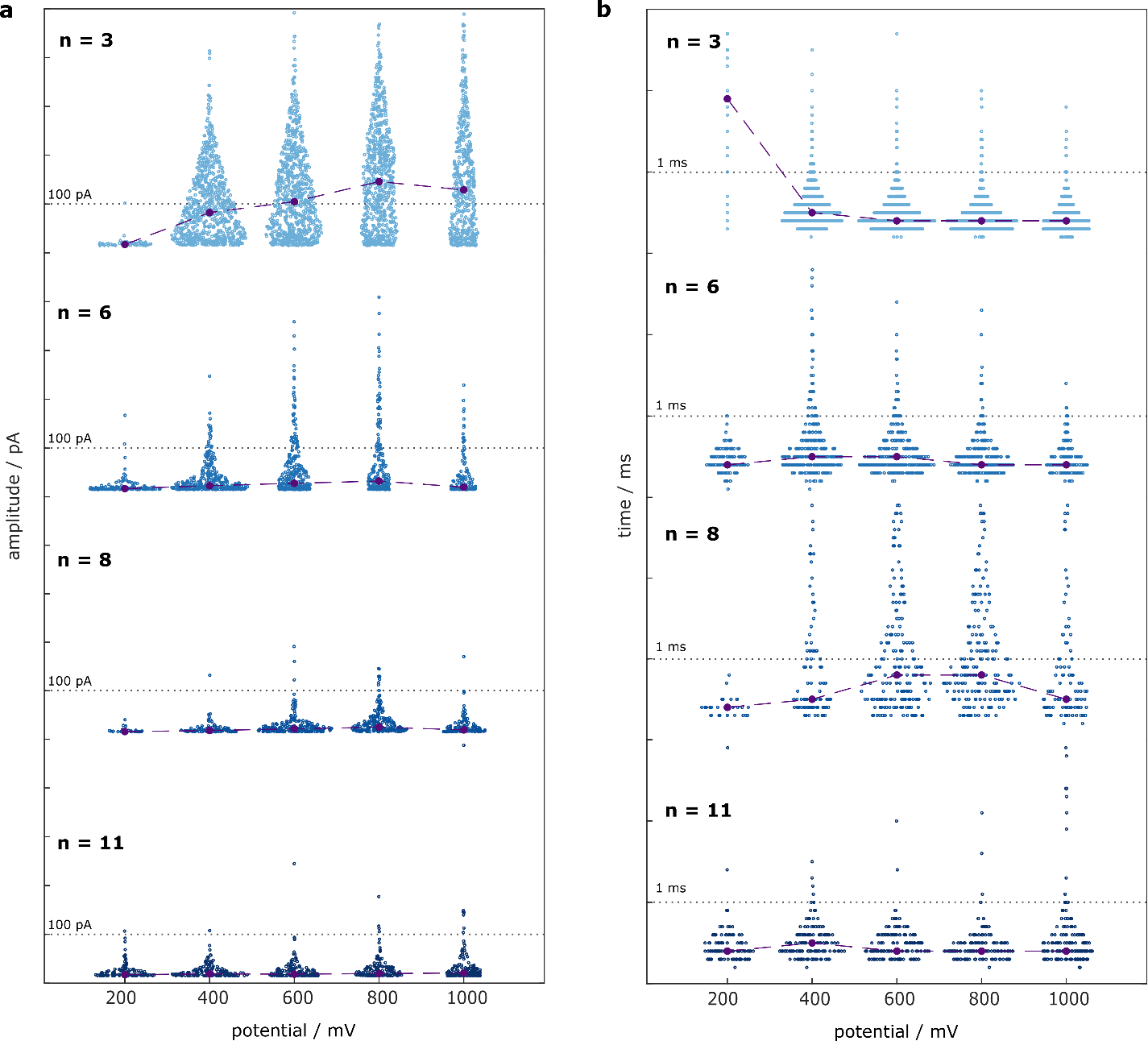


**Figure S5** Distributions of (a) maximum current amplitudes and (b) durations for an impact experiment with 30pM of 20 nm-sized nanoparticles with different shells (COOH-terminated, chain length n ϵ {3,6,8,11}) immersed in 30mM KCl solution for a stepwise-increasing potential from 200 mV to 1000 mV. Each potential was applied for 30s. The median is depicted in purple.

# Partial Contamination of the Electrode During the Experiment

We performed additional impact experiments under buffer conditions supporting a fast oxidation process to investigate the effect of electrode oxidation. To this end, we used 30 pM of 40 nm particles with chain length n=6, -COOH terminated in 100 mM KCl at pH12 and studied the interfering mechanism of oxide formation by comparing two experiments where the electrolyte was either previously purged with N_2_ to remove dissolved oxygen or was untreated. The results in Figure S6 confirm a significant influence of electrode oxidation processes.


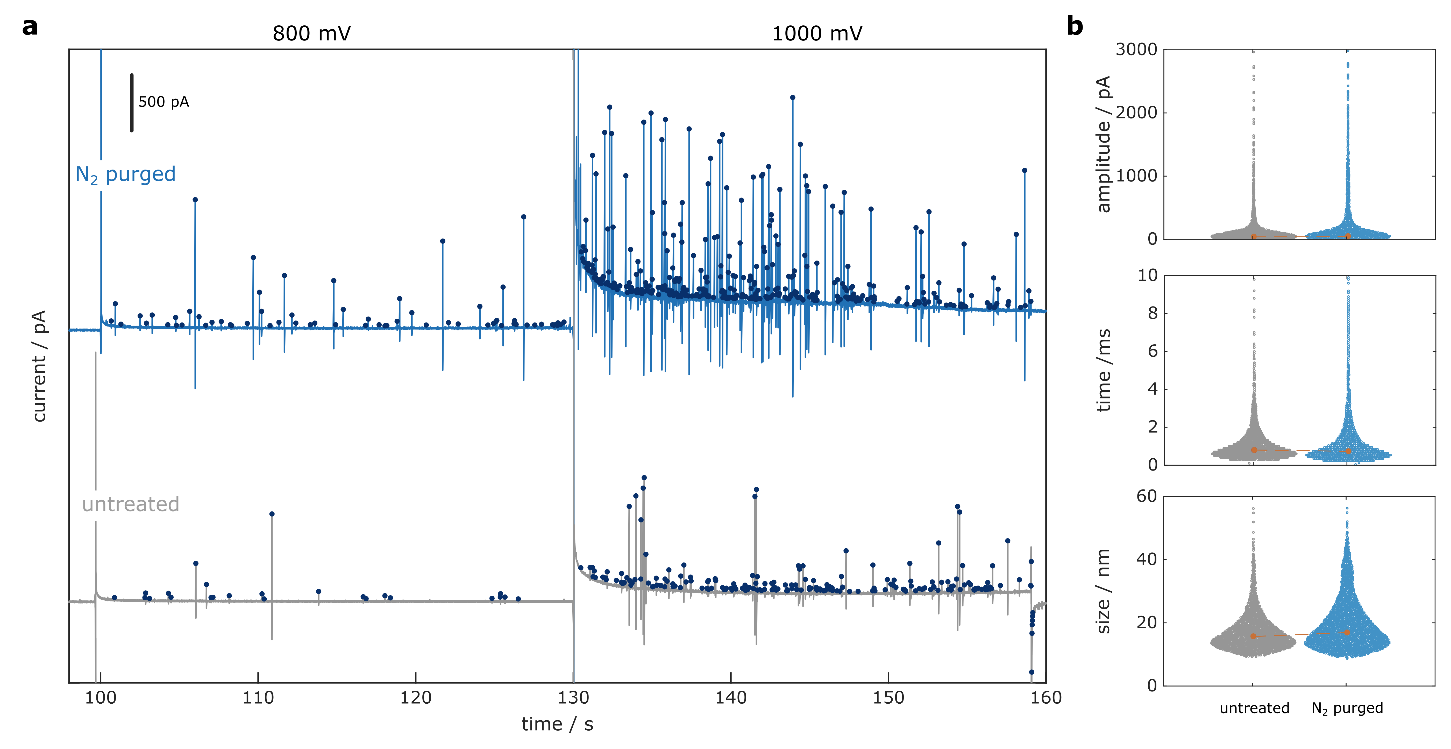
**Figure S6** Ongoing metal-oxide formation affects the experimental outcome. The nitrogen-purged solution leads to current spikes with higher amplitudes and slightly longer durations, both supporting that more charge is transferred upon impact. (a) Exemplary raw current traces for an impact study in N_2_-purged and untreated electrolyte. (b) Current amplitudes, durations, and associated particle sizes for a bias potential of 800 mV vs. Ag/AgCl. The median is shown in orange.

# Transient Current Dips Preceding Oxidation Peaks

**Table S1** Percentage of collisions with preceding current dip. The threshold for the drop in current was $\left| \Delta i_{\text{pk2pk}} \right|=15 \text{pA}$.

|  | applied potential | | | | |
| --- | --- | --- | --- | --- | --- |
| ligand length | 200 mV | 400 mV | 600 mV | 800 mV | 1000 mV |
| n = 3 | 3.6% | 0.2% | 0.0% | 0.2% | 0.3% |
| n = 6 | 2.5% | 1.8% | 1.1% | 1.2% | 1.4% |
| n = 8 | 2.9% | 0.0% | 0.5% | 0.4% | 0.7% |
| n = 11 | 5.2% | 1.0% | 0.9% | 1.3% | 1.4% |

**Table S2** Mean amplitude of the positive oxidation current for all peaks with preceding current dip.

|  | applied potential | | | | |
| --- | --- | --- | --- | --- | --- |
| ligand length | 200 mV | 400 mV | 600 mV | 800 mV | 1000 mV |
| n = 3 | 59.1 pA | 65.6 pA | - | 20.3 pA | 37.1 pA |
| n = 6 | 25.0 pA | 25.4 pA | 23.1 pA | 21.3 pA | 16.9 pA |
| n = 8 | 18.3 pA | - | 16.2 pA | 15.4 pA | 16.7 pA |
| n = 11 | 22.1 pA | 21.2 pA | 19.2 pA | 18.8 pA | 54.6 pA |

**Table S3** Mean amplitude of the positive oxidation current for all peaks without preceding current dip.

|  | applied potential | | | | |
| --- | --- | --- | --- | --- | --- |
| ligand length | 200 mV | 400 mV | 600 mV | 800 mV | 1000 mV |
| n = 3 | 17.9 pA | 98.2 pA | 129.3 pA | 165.5 pA | 139.7 pA |
| n = 6 | 22.4 pA | 41.4 pA | 60.9 pA | 73.7 pA | 50.1 pA |
| n = 8 | 17.8 pA | 25.1 pA | 27.8 pA | 34.4 pA | 26.4 pA |
| n = 11 | 24.4 pA | 25.0 pA | 25.7 pA | 26.7 pA | 31.1 pA |


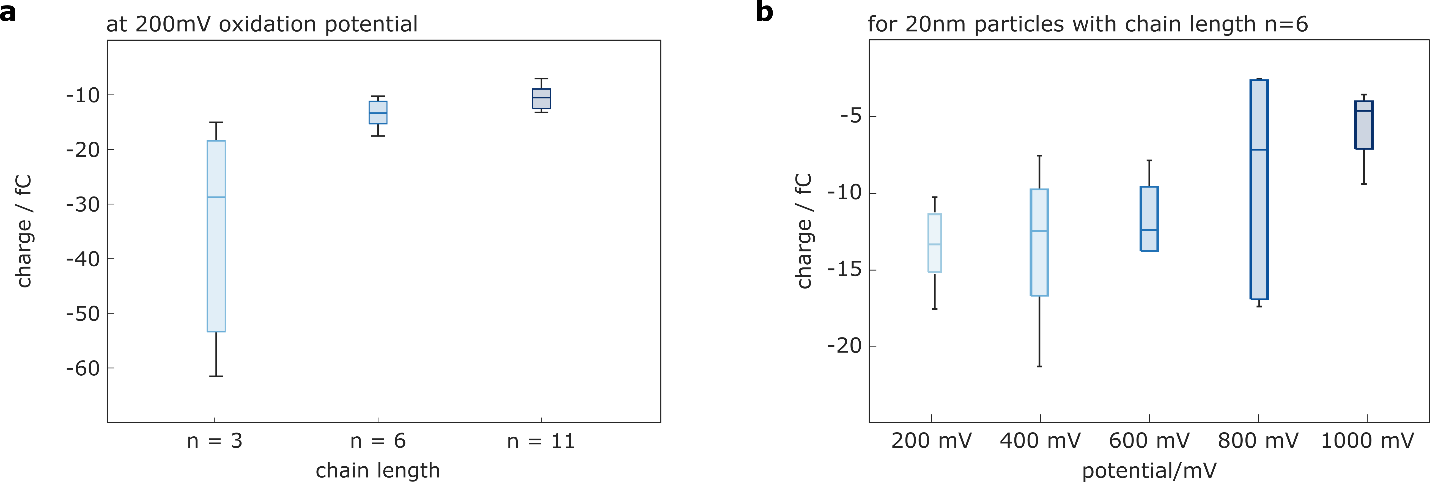


**Figure S7** Normalized charge of preceding current dip measured from 20 nm-sized particles, COOH-terminated (a) at a bias potential of 200 mV for different ligand lengths and (b) for a chain length of n=6 at different applied oxidation potentials from 200 mV to 1000 mV. The differential capacitance can be extracted from the slope in Figure S7a and is ~9 fF for the 20 nm-sized particles with n=6, COOH-terminated.^[36]^ The specific capacitance is deduced from the differential capacitance divided by the surface area of the particle, assumed to be $A=4\pi r^{2}=1256 \text{n}\text{m}^{\text{2}}=1.256\times{10}^{-11}\text{ c}\text{m}^{\text{2}}$, leading to a value of on the order of $700 \mu\text{F}/\text{c}\text{m}^{\text{2}}$.

# Exemplary Current Traces for Different Particle Variants


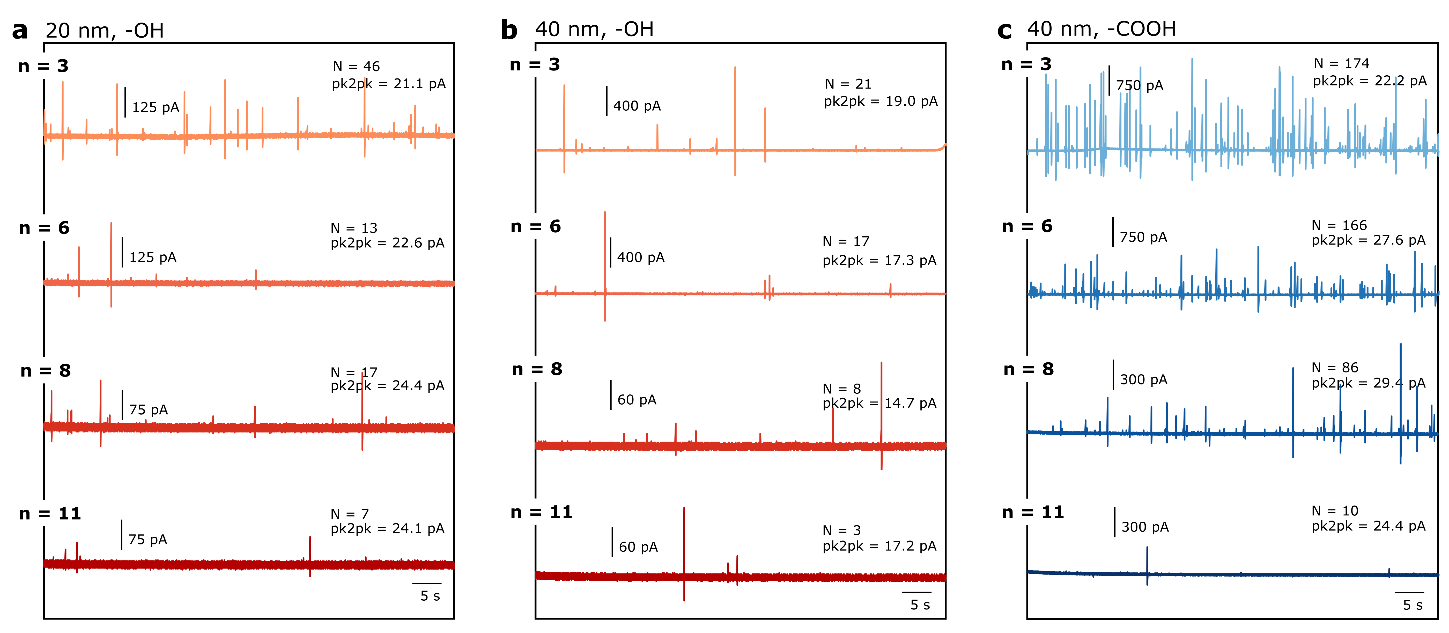


**Figure S8** **Exemplary current traces** for impact studies in 30mM KCl solution and a bias potential of 800 mV vs. Ag/AgCl with 30 pM of (a) 20 nm-sized particles with -OH moiety, (b) 40 nm-sized particles with -OH-moiety and (c) 40 nm-sized particles with -COOH-moiety.

**Table S4** 3-way ANOVA test on the impact frequencies shown in Figure 4. The significance level is indicated by * (p < 0.05), ** (p < 0.01) and *** (p < 0.001).

| effect | Sum. Sq. | df | Mean Sq. | F | p-value | Sig. |
| --- | --- | --- | --- | --- | --- | --- |
| ligand length | 18.34 | 3 | 6.11 | 52.70 | 8.19e-26 | *** |
| diameter | 0.57 | 1 | 0.57 | 4.94 | 0.0279 | * |
| end group | 13.05 | 1 | 13.05 | 112.48 | 1.48e-21 | *** |
| ligand length / diameter | 0.66 | 3 | 0.22 | 1.89 | 0.13 |  |
| ligand length / end group | 9.73 | 3 | 3.24 | 27.95 | 2.15e-15 | *** |
| diameter / end group | 13.26 | 1 | 13.26 | 114.29 | 8.04e-22 | *** |
| ligand length / diameter / end group | 6.00 | 3 | 2.00 | 17.25 | 4.12e-10 | *** |
| error | 25.98 | 224 | 0.12 |  |  |  |
| total | 87.59 | 239 |  |  |  |  |

To evaluate the statistical significance of the findings presented in Figure 4, we performed a 3-way ANOVA examining the effects of ligand length, particle size, and functional end group. The significance levels are denoted by * (p < 0.05), ** (p < 0.01), and *** (p < 0.001). Significant main effects were observed for the ligand length (***), the end group (***) and the particle size (*). However, additional strong interactions were identified between ligand length and end group (***), diameter and end group (***), and among all three variables (***). Thus, our results highlight the complexity of the particle-electrode interactions, demonstrating that the impact frequency depends not only on individual factors but also on the interplay between molecular structure, particle size, and surface chemistry.

# Peak Shapes for 20 nm-Sized Particles with OH-Terminal for Increasing Potentials


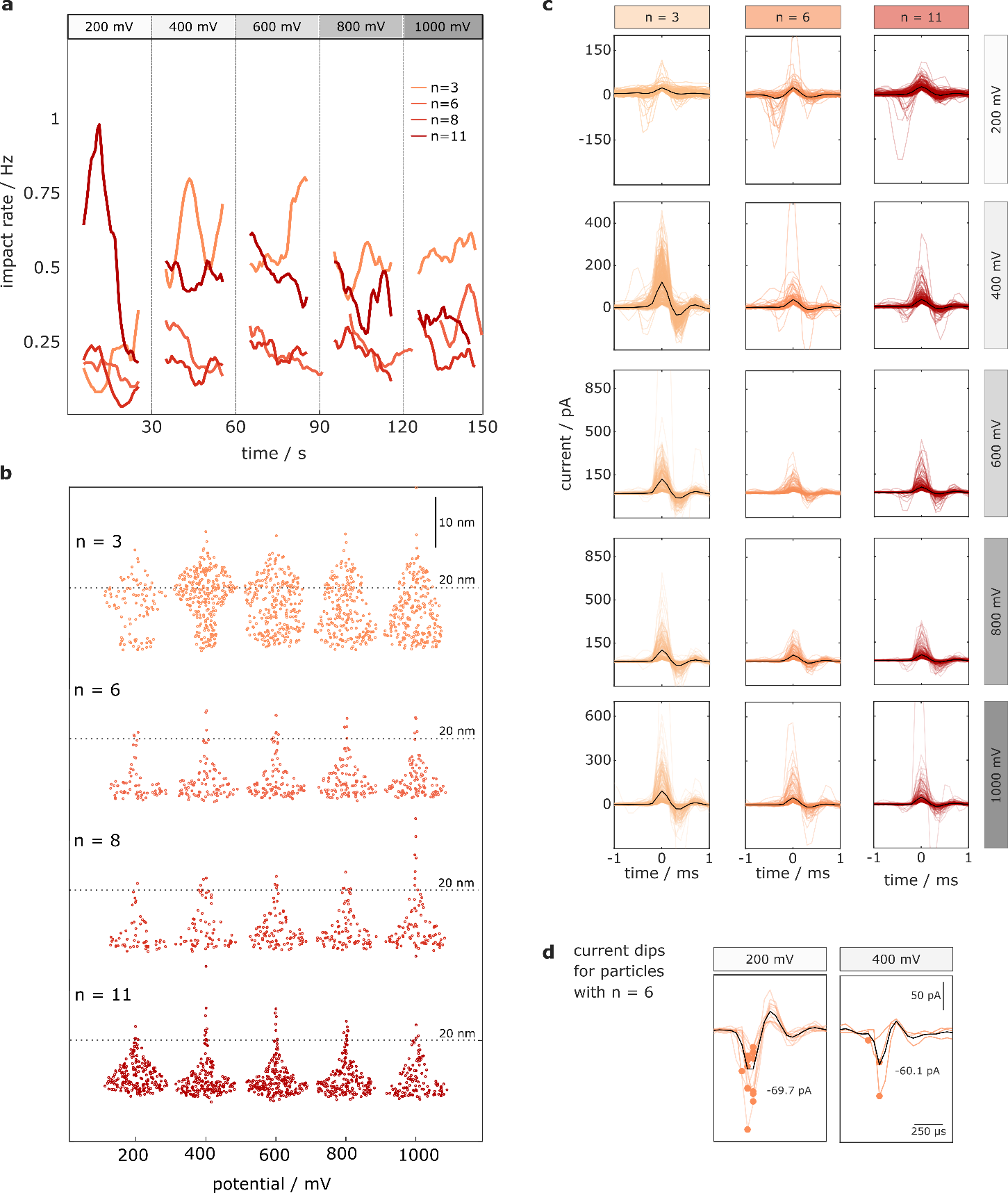


**Figure S9** Effect of applied oxidation potential on the collision rate for OH-terminated 20 nm-sized particles. The impacts are measured from 30 pM particles immersed in 30 mM KCl. (a) Temporal evolution of the mean impact rate based on the recordings of 15 electrodes. (b) Distributions of the estimated particle sizes for different oxidation potentials. (c) Overlay of all recorded current peaks for selected variants. The black line shows the mean transient behavior. (d) Overlay of current peaks with a preceding current dip >|15pA| for particles with chain length n=6 at increasing bias potentials. The values indicate the averaged negative current amplitude. The average duration of the dips ranges between 380 µs and 450 µs.

# Associated Injected Charge to Figure 4 and Figure 5


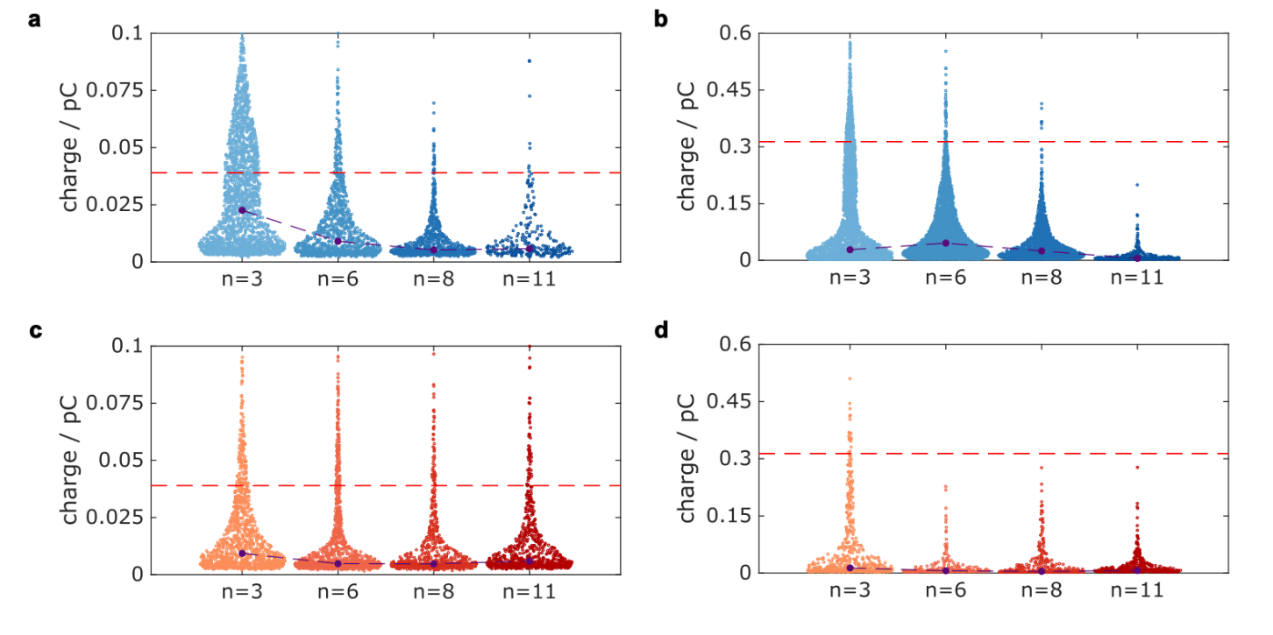


**Figure S10** **Associated injected charges** for impact studies in 30 mM KCl solution and a bias potential of 800 mV vs. Ag/AgCl with 30 pM of (a) 20 nm-sized particles with -COOH moiety, (b) 40 nm-sized particles with -COOH-moiety and (c) 20 nm-sized particles with -OH-moiety and (d) 40 nm-sized particles with -OH moiety. The red lines indicate the theoretical charge assuming a full particle oxidation upon collision.

# Effect of Electrolyte Composition on the Particle Redox Activity

### Colloidal Stability of Particles in 100 mM KCl

**
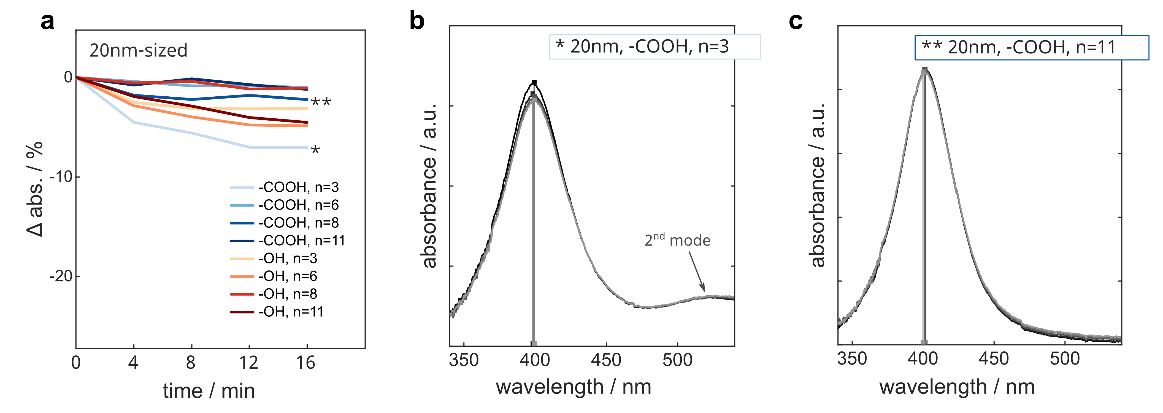
**

**Figure S11** Colloidal stability of modified nanoparticles in 100 mM KCl solution. (a) Relative change in maximum UV/Vis absorbance for all 20 nm-sized particle variants. (b) Exemplary successive UV/Vis recordings of particles with a mercaptopropionic acid corona (-COOH, n=3) and (c) with a mercaptoundecanoic acid corona (-COOH, n=11) within the timeframe of 15 min.

### Recording in 30mM KCl at Various pH Values


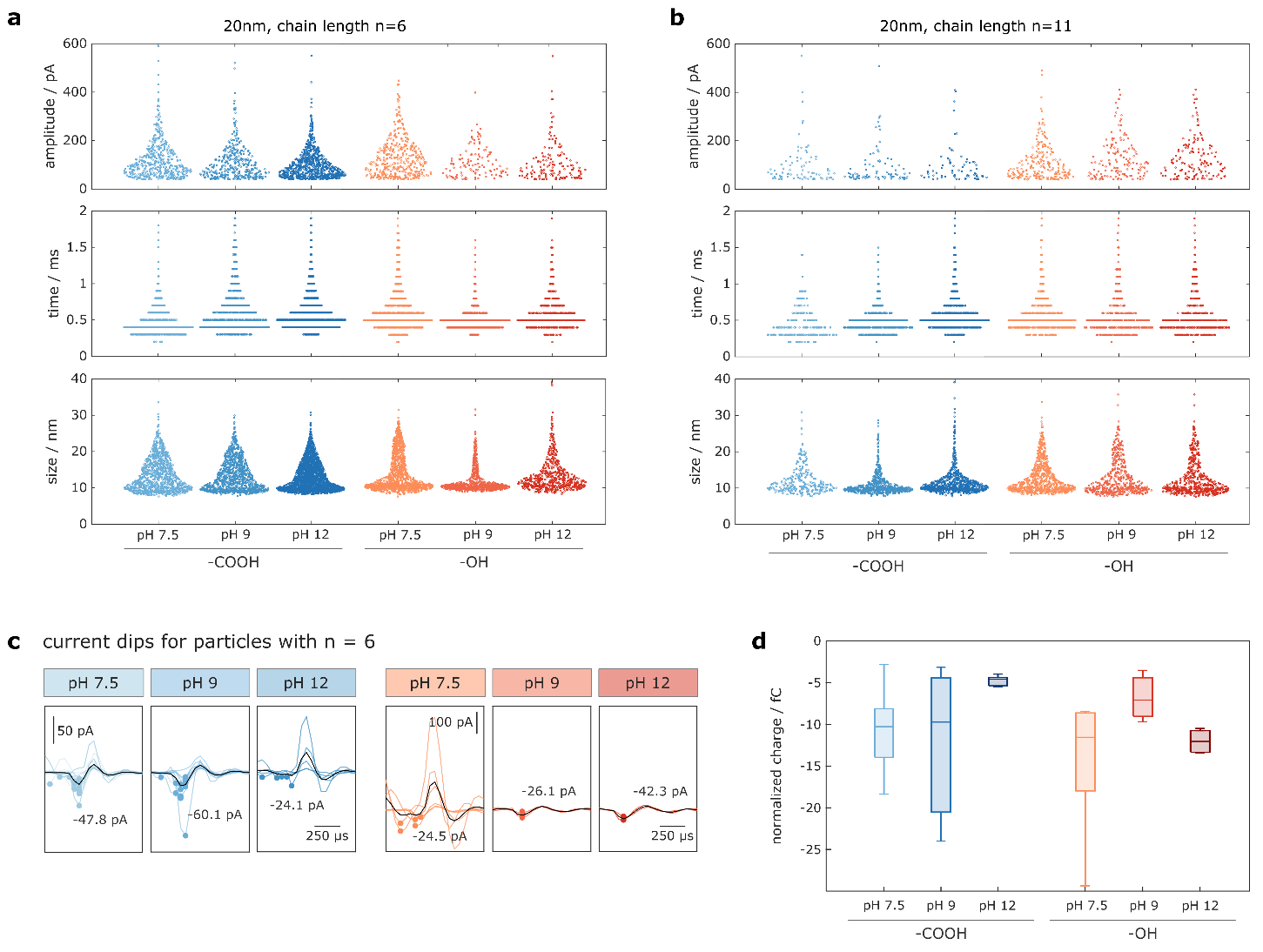


**Figure S12** Statistical data to impact experiments at different pH shown in Figure 6. (a) Maximum current amplitudes, durations, and associated sizes for particles with chain length n=6 and (b) n=11 in 30 mM KCl solution at different pH for a constant applied potential of 800 mV vs. Ag/AgCl.

### Impact Experiments in Modified Phosphate Buffered Saline


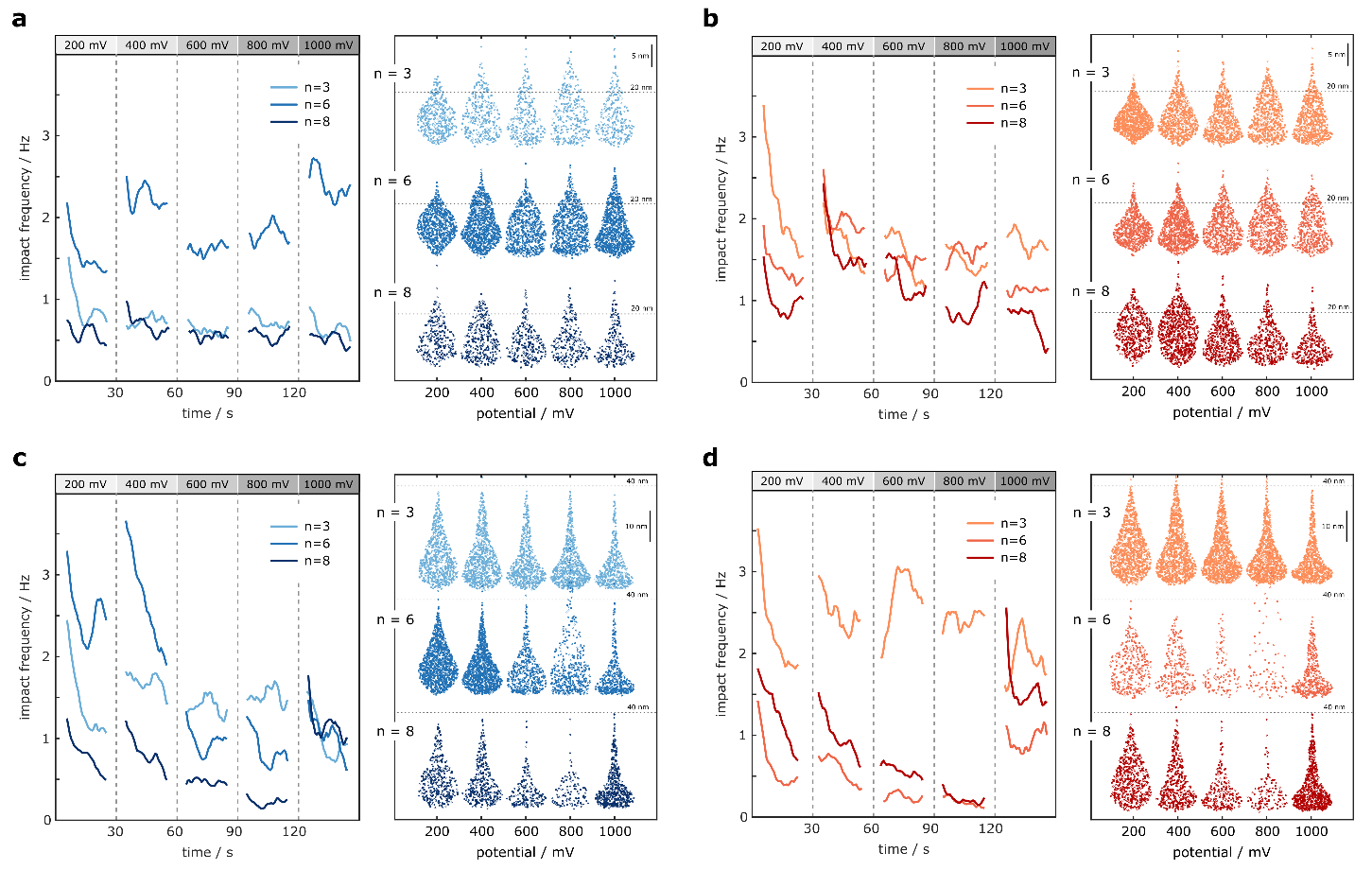


**Figure S13** Recordings in phosphate-buffered saline for exemplary particle species at increasing applied potentials for (a) 20 nm-sized particles COOH-terminated, (b) 20 nm-sized particles OH-terminated, (c) 40 nm-sized particles COOH-terminated, and (d) 40 nm-sized particles OH-terminated. The left graphs depict the mean impact frequency of 15 channels and the right graphs show the associated particle size distributions based on all considered peaks of the experiment. In all experiments, 30 pM particles were immersed in 1x modified Dulbecco’s phosphate buffered saline and the potential was successively stepped from 200 mV to 1000 mV every 30 s.

# Effect of a Mixed Monolayer Particle Corona on Redox Activity

Figure S14 illustrates an improved redox activity of biotin-decorated particles by using a co-assembly with unspecific alkanethiol molecules (n=3, -COOH) as spacers.


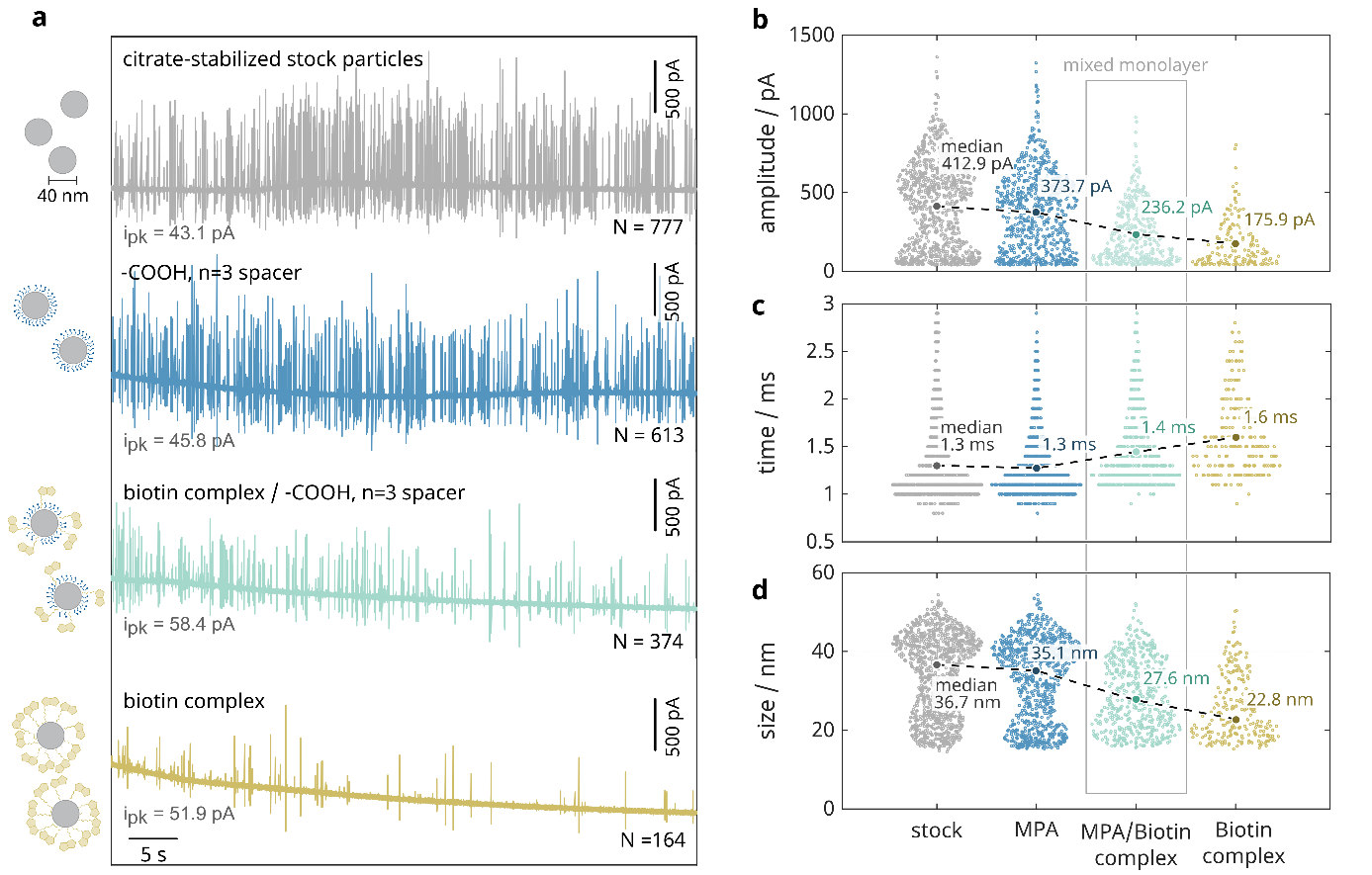


**Figure S14** Single impacts of biotin-functionalized 40 nm-sized silver nanoparticles measured in 25mM KCl and a bias potential of 800 mV vs. Ag/AgCl. (a) Raw current traces (i) for commercially available citrate-stabilized particles, (ii) mercaptopropionic acid-modified silver nanoparticles (-COOH, n=3), (iii) particles with a mixed monolayer of -COOH, n=3 and a protruding alkanethiol-biotin molecule, (iv) and nanoparticles carrying only the specific alkanethiol-biotin complex. The particle concentration was ~45pM in each experiment. Statistical analysis of the current transients including swarm plots of the (b) distribution of maximum currents, (c) time duration of the impacts and (d) corresponding particle sizes obtained from the injected charge. Additionally, the median values are indicated in panels (b) to (d).

As a model system, we modified 40 nm-sized silver nanoparticles either by a co-assembly of a protruding ~2.5 nm-long alkanethiol-biotin complex and short mercaptopropionic acid (n=3, -COOH) molecules as spacers or exclusively with biotin complexes. The complex ligand was created via EDC/s-NHS click chemistry of mercaptopropionic acid and pentylamine-biotin.^[3]^ Prior to impact studies, we verified the successful functionalization via UV/vis recordings and zeta-potential measurements in 25 mM KCl. The presence of biotin on the particle shell was confirmed via binding to streptavidin-horseradish peroxidase (HRP) and a subsequent TMB test causing a color change.

# References

[1] X. Zhang, M. R. Servos, J. Liu, *Chem. Commun.* **2012**, *48*, 10114.

[2] X. Zhang, M. R. Servos, J. Liu, *J. Am. Chem. Soc.* **2012**, *134*, 7266.

[3] L. J. K. Weiß, P. Rinklin, B. Thakur, E. Music, H. Url, I. Kopic, D. Hoven, M. Banzet, T. von Trotha, D. Mayer, B. Wolfrum, *ACS Sens.* **2022**, *7*, 1967.

[4] A. Heuer-Jungemann, N. Feliu, I. Bakaimi, M. Hamaly, A. Alkilany, I. Chakraborty, A. Masood, M. F. Casula, A. Kostopoulou, E. Oh, K. Susumu, M. H. Stewart, I. L. Medintz, E. Stratakis, W. J. Parak, A. G. Kanaras, *Chem. Rev.* **2019**, *119*, 4819.

[5] T. T. Ehler, N. Malmberg, L. J. Noe, *J. Phys. Chem. B* **1997**, *101*, 1268.

[6] M. D. Malinsky, K. L. Kelly, G. C. Schatz, R. P. Van Duyne, *J. Am. Chem. Soc.* **2001**, *123*, 1471.

[7] A. S. Dileseigres, Y. Prado, O. Pluchery, *Nanomaterials* **2022**, *12*, 292.

[8] D. Roy, J. Fendler, *Advanced Materials* **2004**, *16*, 479.

[9] N. Camillone III, C. E. D. Chidsey, G. Liu, T. M. Putvinski, G. Scoles, *The Journal of Chemical Physics* **1991**, *94*, 8493.

[10] A. Kuzma, M. Weis, S. Flickyngerova, J. Jakabovic, A. Satka, E. Dobrocka, J. Chlpik, J. Cirak, M. Donoval, P. Telek, F. Uherek, D. Donoval, *Journal of Applied Physics* **2012**, *112*, 103531.

[11] M. J. Hostetler, J. J. Stokes, R. W. Murray, *Langmuir* **1996**, *12*, 3604.

[12] Z. Dai, H. Ju, *Physical Chemistry Chemical Physics* **2001**, *3*, 3769.

[13] C. Vericat, M. E. Vela, R. C. Salvarezza, *Phys. Chem. Chem. Phys.* **2005**, *7*, 3258.

[14] G. Yang, N. A. Amro, Z. B. Starkewolfe, G. Liu, *Langmuir* **2004**, *20*, 3995.

[15] L. Srisombat, A. C. Jamison, T. R. Lee, *Colloids and Surfaces A: Physicochemical and Engineering Aspects* **2011**, *390*, 1.

[16] J. B. Schlenoff, M. Li, H. Ly, *J. Am. Chem. Soc.* **1995**, *117*, 12528.

[17] P. Kr. Ghorai, S. C. Glotzer, *J. Phys. Chem. C* **2007**, *111*, 15857.

[18] M. D. Scanlon, P. Peljo, M. A. Méndez, E. Smirnov, H. H. Girault, *Chem. Sci.* **2015**, *6*, 2705.

[19] T. Djebaili, J. Richardi, S. Abel, M. Marchi, *J. Phys. Chem. C* **2013**, *117*, 17791.

[20] R. G. Nuzzo, L. H. Dubois, D. L. Allara, *J. Am. Chem. Soc.* **1990**, *112*, 558.

[21] J. P. Folkers, P. E. Laibinis, G. M. Whitesides, J. Deutch, *J. Phys. Chem.* **1994**, *98*, 563.

[22] C. S. Weisbecker, M. V. Merritt, G. M. Whitesides, *Langmuir* **1996**, *12*, 3763.

[23] C. Weeraman, A. K. Yatawara, A. N. Bordenyuk, A. V. Benderskii, *J. Am. Chem. Soc.* **2006**, *128*, 14244.

[24] E. Heikkilä, A. A. Gurtovenko, H. Martinez-Seara, H. Häkkinen, I. Vattulainen, J. Akola, *J. Phys. Chem. C* **2012**, *116*, 9805.

[25] D. S. Bolintineanu, J. M. D. Lane, G. S. Grest, *Langmuir* **2014**, *30*, 11075.

[26] C. Fillafer, M. Wirth, F. Gabor, *Langmuir* **2007**, *23*, 8699.

[27] C. M. Maguire, M. Rösslein, P. Wick, A. Prina-Mello, *Science and Technology of Advanced Materials* **2018**, *19*, 732.

[28] P. J. Moncure, J. E. Millstone, J. E. Laaser, *J. Phys. Chem. B* **2023**, *127*, 9366.

[29] G. E. Poirier, M. J. Tarlov, H. E. Rushmeier, *Langmuir* **1994**, *10*, 3383.

[30] L. Ramin, A. Jabbarzadeh, *Langmuir* **2011**, *27*, 9748.

[31] J. C. Love, L. A. Estroff, J. K. Kriebel, R. G. Nuzzo, G. M. Whitesides, *Chem. Rev.* **2005**, *105*, 1103.

[32] M. Baghbanzadeh, F. C. Simeone, C. M. Bowers, K.-C. Liao, M. Thuo, M. Baghbanzadeh, M. S. Miller, T. B. Carmichael, G. M. Whitesides, *J. Am. Chem. Soc.* **2014**, *136*, 16919.

[33] M. D. Porter, T. B. Bright, D. L. Allara, C. E. D. Chidsey, *J. Am. Chem. Soc.* **1987**, *109*, 3559.

[34] C. E. D. Chidsey, D. N. Loiacono, *Langmuir* **1990**, *6*, 682.

[35] E. Cooper, G. J. Leggett, *Langmuir* **1999**, *15*, 1024.

[36] M. Azimzadeh Sani, N. G. Pavlopoulos, S. Pezzotti, A. Serva, P. Cignoni, J. Linnemann, M. Salanne, M.-P. Gaigeot, K. Tschulik, *Angewandte Chemie* **2022**, *134*, e202112679.
